# Morphological, genotypic and metabolomic signatures confirm interfamilial hybridization between the ubiquitous kelps *Macrocystis* (Arthrothamnaceae) and *Lessonia* (Lessoniaceae)

**DOI:** 10.1038/s41598-020-65137-3

**Published:** 2020-05-19

**Authors:** Pedro Murúa, RuAngelie Edrada-Ebel, Liliana Muñoz, Sylvia Soldatou, Nathalie Legrave, Dieter G. Müller, David J. Patiño, Pieter can West, Frithjof C. Küpper, Renato Westermeier, Rainer Ebel, Akira F. Peters

**Affiliations:** 1 Instituto de Acuicultura, Universidad Austral de Chile, Sede Puerto Montt, PO box 1327 Puerto Montt, Chile; 2 The Scottish Association for Marine Science, Scottish Marine Institute, Culture Collection for Algae and Protozoa, Oban, Argyll, PA37 1QA Scotland, United Kingdom; 3 Aberdeen Oomycete Group, College of Life Sciences and Medicine, University of Aberdeen, Foresterhill, AB25 2ZD Aberdeen, United Kingdom; 4 The Natural Products Metabolomics Group, Strathclyde Institute of Pharmacy and Biomedical Sciences, University of Strathclyde, The John Arbuthnott Building, 161 Cathedral Street, Glasgow, G4 0RE United Kingdom; 5 Marine Biodiscovery Centre, Department of Chemistry, University of Aberdeen, Meston Building, Meston Walk, Old Aberdeen, AB24 3UE United Kingdom; 6 Fachbereich Biologie der Universität Konstanz, D-78457 Konstanz, Germany; 7 School of Biological Sciences, University of Aberdeen, Cruickshank Building, St Machar Drive, Aberdeen, AB24 3UU Scotland UK; 8 Bezhin Rosko, 40 rue des pêcheurs, 29250 Santec, Brittany France

**Keywords:** Ecology, Genetics, Evolution

## Abstract

*Macrocystis pyrifera* and *Lessonia spicata* are economically and ecologically relevant brown seaweeds that recently have been classified as members of two separated families within Laminariales (kelps). Here we describe for the first time the *Macrocystis pyrifera* x *Lessonia spicata* hybridization in the wild (Chiloe Island, Southeastern Pacific), where populations of the two parents exist sympatrically. Externally, this hybrid exhibited typical features of its parents *M. pyrifera* (cylindrical and flexible distal stipes, serrate frond margins and presence of sporophylls) and *L. spicata* (rigid and flat main stipe and first bifurcation), as well as intermediate features between them (thick unfused haptera in the holdfast). Histological sections revealed the prevalence of mucilage ducts within stipes and fronds (absent in *Lessonia*) and fully developed unilocular sporangia in the sporophylls. Molecular analyses confirmed the presence of the two parental genotypes for ITS1 nrDNA and the *M. pyrifera* genotype for two predominantly maternally inherited cytoplasmic markers (COI and *rbc*LS spacer) in the tissue of the hybrid. A metabolome-wide approach revealed that this hybrid is more chemically reminiscent to *M. pyrifera*. Nevertheless, several hits were identified as *Lessonia* exclusive or more remarkably, not present in any of the parent. Meiospores developed into apparently fertile gametophytes, which gave rise to F1 sporophytes that reached several millimeters before suddenly dying. *In-vitro* reciprocal crossing of Mar Brava gametophytes from both species revealed that although it is rare, interfamilial hybridization between the two species is possible but mostly overcome by pseudogamy of female gametophytes.

## Introduction

Brown algae from the order Laminariales (a.k.a. kelps) are among the most conspicuous organisms in cold-temperate marine regions in both hemispheres. They form forest-like communities that make up most of the biomass in such ecosystems, and are of paramount importance as breeding grounds and nurseries, substrata and food for many marine species^1,2^. Kelps globally account for 173 TgC yr^−1^ carbon sequestration worldwide^3^, they have a key role in the global iodine cycle and bear variable tolerance under current climate change scenarios^4,5^. Commercially, they have been harvested for food and feed applications as well as soil fertilization, bioremediation processes, metabolite extraction and recently, bioconversion to second-generation biofuels^6,7^. Within kelps, the giant kelp *Macrocystis* (family Arthrothamnaceae) is one of the top candidates for brown algal mariculture and domestication in the Pacific due to its remarkable productivity, the highest for marine macroalgae^8,9^. *Lessonia* (Lessoniaceae), on the other hand, is commonly harvested for raw material for alginate manufacturing and abalone feed, and nowadays intensely exploited in northern Chile^10,11^.

**Figure 1:**
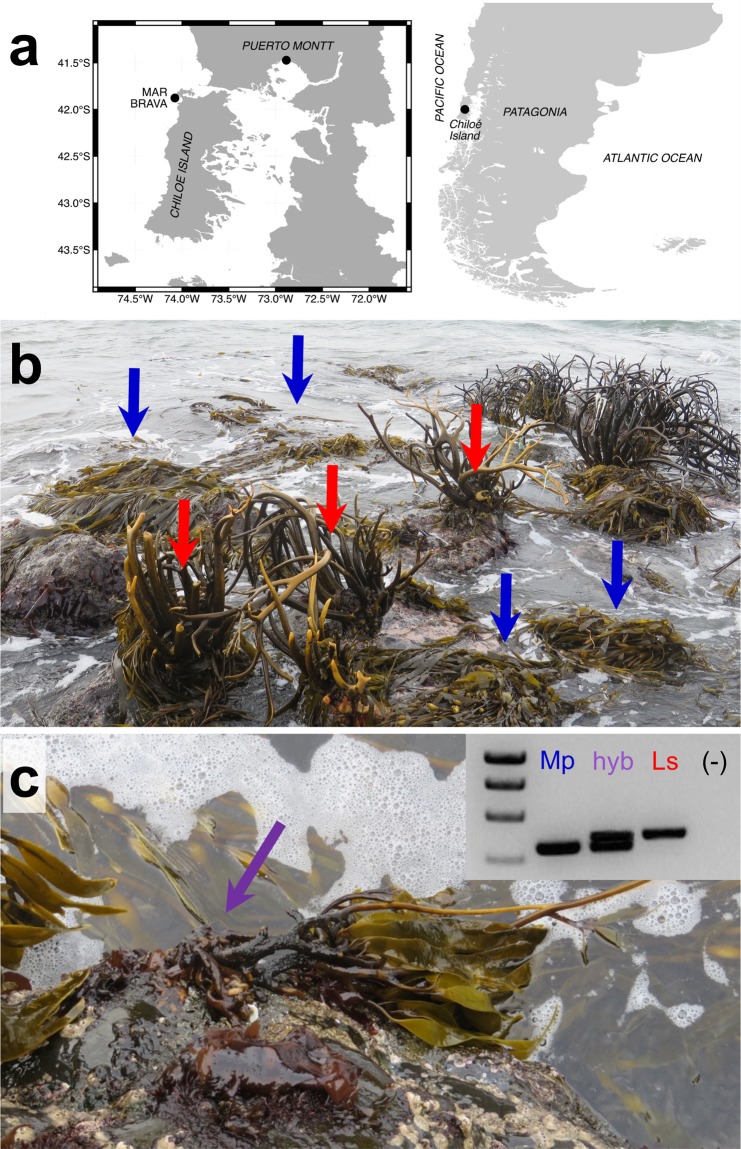
Wild interfamilial *Macrocystis* x *Lessonia* specimen. (**a**) Geographical location of the sympatric *M. pyrifera* and *L. spicata* in Mar Brava, Chiloe Island, Chile. (**b**) Representative landscape of the *Macrocystis* (blue arrow) and *Lessonia* (red arrow) assemblages in the intertidal of Mar Brava. (**c**) Full morphology of the hybrid *Macrocystis* x *Lessonia* in the field, pointing out the holdfast with unfused-thick haptera (purple arrow). Inset: ITS1 amplicon pattern on an electrophoresis gel for *M. pyrifera* (Mp), *L. spicata* (Lp) and the hybrid (hyb) from this locality. (−): negative control.

Ecologically, *M. pyrifera* and *L. spicata* are dominant kelps in terms of cover and biomass and key bioengineers in their habitats. Particularly in the Southeastern Pacific, *M. pyrifera* occurs from Peru to the southern end of Chile in Tierra del Fuego, encompassing submarine habitats of two well-defined biogeographic provinces with considerably different environmental conditions^12,13^. For several years, *Macrocystis* was separated in two sister species (the northern *M. integrifolia*, and southern *M. pyrifera;* Hoffmann & Santelices, 1997), based on solid morphological and developmental distinctions (i.e. holdfast morphology, size, growth rates and patterns). More recently, molecular and biological data have confirmed conspecificity of these two taxa^14–16^, and we are increasingly using the term “morph” to name the two different forms. The *Lessonia nigrescens* complex, on the other hand, is now under a taxonomic review in northern Patagonia, since populations from northern and central-austral Chile were different cryptic species and were re-classified as *L. berteroana* and *L. spicata*, respectively^17–19^. While *L. spicata* typically inhabits high-energy zones in the wave-exposed intertidal, *M. pyrifera* has higher abundance in the subtidal. Nonetheless, the giant kelp may colonize intertidal areas, exhibiting significant differences in growth and reproduction cycles in order to tackle such harsh conditions^20^. Sometimes, *M. pyrifera* and *L. spicata* may form assemblages in southern Chile, showing sympatry in the lower eulittoral zone^21^ (Fig. 1).

One of the most accepted definitions for hybridization refers to the interbreeding of individuals from two distinct populations or groups of populations, where individuals must be distinguishable on the basis of one or more heritable characters (see Harrison and Larson^22^ and references therein). In animals exceptionally, species are able to hybridize, including hominids like Neanderthals and modern humans in Eastern Europe 40,000 years ago^23^. In higher plants, hybridization is pervasive, contributing actively to speciation. It is also artificially manipulated to get higher quality crops^24,25^. Hybridization normally changes the genetic variability among/within populations through gene introgression but may even lead to extinction vortexes or the generation of invasive genotypes^25^. In seaweeds, the role of hybridization is still not clearly understood but has been reported in a few members of Ectocarpales, Laminariales and Fucales^26–28^ and also red algae^29^. Recent studies started to highlight its participation in habitat adaptation, especially in low-dispersal *Fucus* species^26,30^. More importantly, it seems that for several species - like the model brown alga *Ectocarpus* - hybridization is normally going on in a proportion of the progeny, but in most cases introgression is strongly restricted by post-zygotic barriers^31^.

The closer the phylogenetic relationship, the more common the hybridization is. Interfamilial hybridization is on a general rule very rare. Particularly in seaweeds, the first and only putative interfamilial hybrid confirmed by both molecular and morphological data corresponds to an *Alaria marginata* x *Lessoniopsis littoralis* specimen^32^. However, after further studies by Lane *et al*.^33^ and later confirmed by transcriptomics^34^ and organellar phylogenomics^35,36^ both species belong to the same family Alariaceae. In such studies, *Lessonia* and *Macrocystis* are robustly nested in phylogenetically distant families. For sympatric *Lessonia* and *Macrocystis*, there is no record of morphological features of genetic hybrids so far. Here, we describe the first record of an interfamilial fertile *Macrocystis*-*Lessonia* hybrid in nature, by combining morphological, developmental, genetic and metabolomic evidence. Our results show that this process is very rare but not impossible in field and culture, supporting the hypothesis of influential post-barrier mechanisms restricting the prevalence of kelp interfamilial hybrids in wild marine forests.

## Materials and methods

### Field sampling

A fertile putative hybrid that phenotypically resembled both *Macrocystis* and *Lessonia* was collected on February 2015 from the rocky intertidal in Mar Brava, Chiloé Island, Southern Chile (41°52′S, 74°01′W; Fig. 1). This specimen was recognized in a contact zone between the two co-existing species in this habitat and corresponded to the first sighting of an individual with an intermediate morphology in more than 25 years of sampling at this locality by our research group. In order to corroborate its hybrid nature, the individual was collected and imaged, and clean biomass from the hybrid and neighbours *M. pyrifera* and *L. spicata* was stored in silica gel and 4% formaldehyde in seawater for further molecular analyses and histological comparisons, respectively. For metabolomics, five individuals from every species and three pseudo-replicates from the putative hybrid (equivalent to the rest of the available biomass) were harvested from Mar Brava during low tide, put in cooling boxes at 8 °C and transported to the laboratory.

### Histological sectioning

We followed the histology workflow detailed in Murúa *et al*.^37^. After the formaldehyde treatment, tissues from the stipes, vegetative and reproductive fronds were dehydrated in graded series of ethanol (70%/95% for 2 hrs. and three series of 100%, 3 hrs. each) and defatted/cleared by a 1:1 xylene:chloroform solution (three times of 1 hr. each) before embedding in paraffin (two baths of 3 hrs each). The resulting blocks were sectioned (5 μm) using a LEICA RM2125RT microtome and stained with toluidine blue at 0.05% by a 15 sec immersion. Micrographs were obtained with an EVOS cell imaging microscope.

### Culture of hybrid progeny

We cultivated the F1 generation after Westermeier *et al*.^38^. Sorus areas on the putative hybrid were cut out with a razor blade and washed thoroughly in fresh water. After 1 min they were blotted dry with a towel, introduced into a sealable polyethylene bag and stored dark in a household refrigerator between 4 and 10 °C. One or two days later, autoclaved natural sea water with Provasoli enrichment (PES^39^) at ≈10 °C was added in quantities amounting roughly to 100 ml per 100 cm^2^ sorus tissue, resulting in a dense golden-coloured suspension of swimming spores after 30–60 min. Gametophyte ontogeny and subsequent bulk fertilization were stimulated at 10 °C, 40–50 μmol photons/m/s white light applied for 12 h/day^40^. When sporophytes had reached 1 mm, they were transferred to gas washing bottles with aeration and mechanical agitation with magnetic stirrers. As controls, we cultivated *M. pyrifera* and *L. spicata* from Mar Brava. Relative growth rates corrected for the lack of traceability of specific individuals (RGRc) were calculated measuring the length of 30–60 individuals when they first appeared in culture (ca. week 8) and 15 sporophytes at the week 27. RGRs were calculated using the differences of the log-transformed final and initial lengths of these sporophytes corrected for the time in culture, but as initial size we considered the average value of the bulk sporophytes measured at week 8. RGRs were compared using a linear model (one-way ANOVA) after fulfilling homoscedasticity (Levene’s test) and normality (Shapiro’s test and qqplot distribution) assumptions.

### Culture studies in reciprocal M. pyrifera x L. spicata crosses from Mar Brava, and evaluation of interfamilial hybridization in-vitro

Clonal (unisexual) gametophytes isolated from Mar Brava in 1997 and 1999 were used as starting point (Supplementary Table 1), and cultures were performed as described in Westermeier *et al*.^40^, in order to produce free-floating sporophytes. Gametophytes of different sex were combined in the same ratio (ca 10-15 mg FW per cross) in 1.5 ml Eppendorf with 300 μl PES and carefully homogenized using plastic pestles. The product was placed in 6 cm Petri dishes with 15 ml full strength PES under the same light and temperature conditions aforementioned for gametogenesis. In total, four crosses were performed (two intraspecific, two interfamilial), combinations that were repeated in six independent occasions. Reproductive success (RS) by means of percentage of embryos per female gametophyte was calculated after two weeks^41^. The RS performance between different crosses was compared using a one-way ANOVA with a significance of p < 0.05, after confirming homoscedasticity and normality assumptions. When sporophytes reached 4-5 mm size, their holdfasts were removed (in order to discard possible gametophyte remnants). This also aided to remove potential chimeric holdfast tissue sometimes present in kelps^42,43^. Single blades were then placed in RNA LATER or CTAB buffer for further DNA extractions.

### Molecular analysis

DNA extractions were carried out using the GENEJET Plant Genomic DNA Purification Kit (THERMO SCIENTIFIC) following the manufacturer’s instructions, with an initial CTAB buffer treatment according to Gachon *et al*.^44^. These extractions were performed in the field specimens found at Mar Brava (one *M. pyrifera*, one *L. spicata* and the putative hybrid), in the parental gametophyte stocks from Mar Brava and in non-apogamic sporophytes from the interfamilial crosses (n = 30 per replicate, three replicates). In the field specimens and parental gametophytes, polymerase chain reactions (PCR) were performed to amplify fragments of plastidal *rbc*LS spacer and mitochondrial COI to identify the maternal DNA origin. The nuclear ribosomal ITS1 was used in field and cultured individuals to confirm presence of both parental genotypes^27^, using the procedures stated in related DNA barcoding studies^33,45–47^. Since nuclear ITS1 product contained double bands in case of the hybrid (Fig. 1c), amplicons were cloned using the CLONEJET PCR cloning kit (THERMO SCIENTIFIC) (see Murúa *et al*.^11^ for cloning protocol details). In addition, we designed species-specific primers for *M. pyrifera* [ITSMPF5 (CCCCGAGAAAGAAGTCCGTT) - 5.8SR5 (TTGTGGGAGCCAAGACATCC)] and *L. spicata* [ITSLSF2 (GTGGAAACTCCCTTGGAGGC) - ITSLSR2 (GAGCTTCCTTCACCCTTCCC)] that were tested in both parental kelp gametophytes and interfamilial progeny {touchdown PCR: D 30 s [95 °C], A 30 s [65 °C - > 55 °C (-1deg/cycle)], E 120 s [72 °C]} to confirm the results from the ITS1 generic markers.

We sequenced the PCR products (Sanger) from the field individuals and parental gametophytes and the resulting chromatograms were manually corrected, checked for quality, trimmed and aligned with GENEIOUS v11^48^. Consensus sequences were produced and imported into an alignment containing available *Macrocystis* and *Lessonia* sequences using MAFTT^49^. The final alignments were manually checked and analysed by using the Randomized Accelerated Maximum likelihood method (RaxML^50^) and PhyML^51^ based on the General time reversible model (1000 bootstraps), Neighbour-Joining^52^ method based on the Tamura-Nei model (1000 boostraps) and Bayesian inference using MrBayes V3.1.6^53^ (settings: chain length 2000, subsample frequency 1000, burn in of 10%), implemented in GENEIOUS. Sequences were deposited in GenBank with accession numbers MT250555–MT250568 and MT253647–MT253654.

### Metabolomic analyses

Selected individuals looked clean and were free of endo-epiphytes. About 100 g FW (blades) per individual were dried with paper towel, weighted and chopped in strips of ca. 0.5 × 2 cm using a sterile razor blade. Biomass was extracted 2 times in 100% methanol using a sonicator for 1 hr. A third extraction was carried out using 100% acetone. The three final solutions were combined, filtered using cotton plugs and solvent-evaporated in a rotatory evaporator water bath at 40 °C. To remove the dried extract from the evaporator, a few drops of methanol were added. The final liquid was put in 50 ml Falcon tubes, air-dried for few days under a fume hood and stored at 5 °C in darkness.

Mass spectrometry analyses were carried out according to Macintyre *et al*.^54^. High resolution mass spectrometric data were obtained using a THERMO SCIENTIFIC MS system (LTQ XL/LTQ ORBITRAP DISCOVERY) coupled to a THERMO SCIENTIFIC HPLC system (ACCELA PDA DETECTOR, ACCELA PDA AUTOSAMPLER, and ACCELA PUMP). The following conditions were used: capillary voltage 45 V, capillary temperature 260 °C, and auxiliary gas flow rate 10–20 arbitrary units, sheath gas flow rate 40–50 arbitrary units, spray voltage 4.5 kV, mass range 100–2000 amu (maximum resolution 30000). For LC/MS; WATERS SUNFIRE C18 analytical HPLC column (5 µm, 4.6 × 150 mm) was used with a mobile phase of 0–100% MeOH over 30 min at a flow rate of 1 ml/min.

Mass spectrometry data were processed using the predefined metabolomics workflow described previously^54^. Raw data were converted to mzml format using the MassConvert tool from ProteoWizard^55^ then imported and processed in MZmine 2.40^56^ using predefined settings to extract the features. The processed data from MZmine were incorporated into the customized Dictionary of Natural Products Library version June 2019 through the built-in Excel macro for peak identification and dereplication. “Hits” were taxonomically filtered, and unidentified peaks were double checked against the MS raw data in Xcalibur 2.2. An algorithm was employed to use the molecular formula data set from these databases for dereplication. The data set was further analyzed using SIMCA V 15.0 (Umetrics, Umeå, Sweden) using the unsupervised statistical analysis method, principal component analysis (PCA) and supervised analysis method Orthogonal partial least squares discrimination analysis (OPLS-DA). Heat maps were plotted using the programming software R (version ×64 2.15.2) using a script in the ggplot2 package^57^. Venn diagrams were generated using BioVenn^58^.

## Results

### Anatomical and histological features

**Figure 2:**
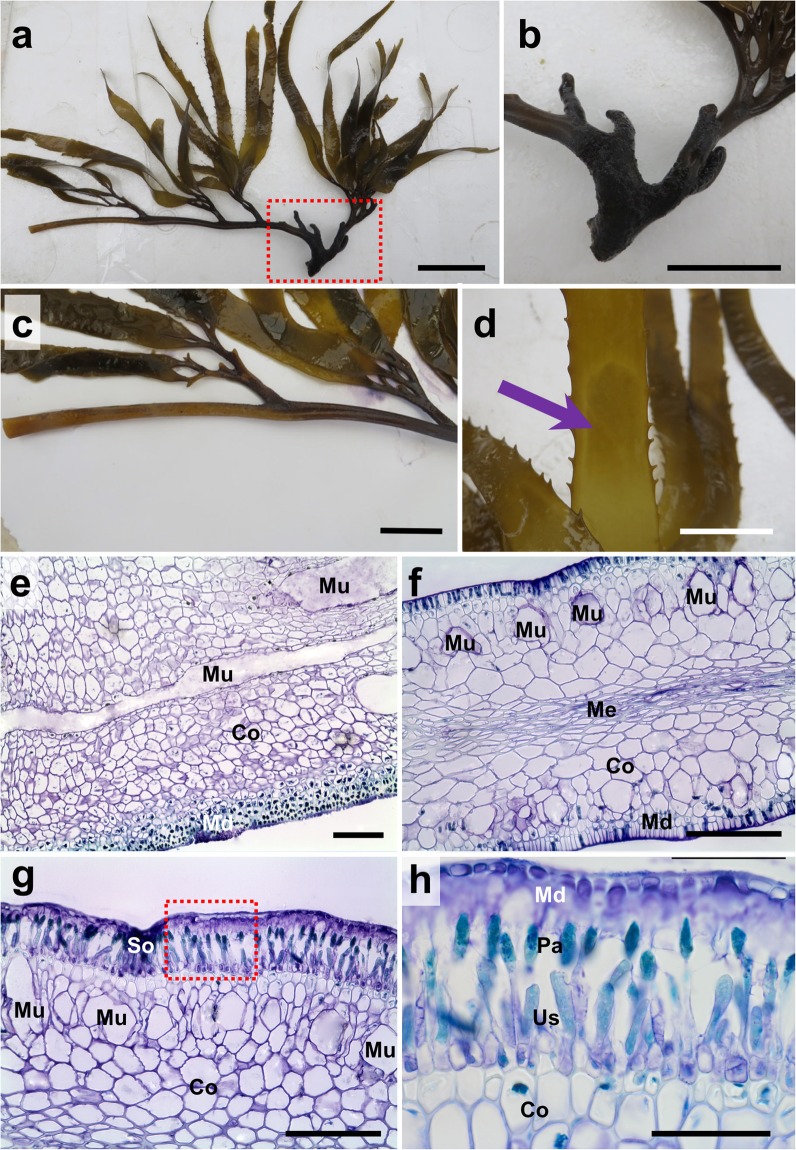
The *Macrocystis* x *Lessonia* hybrid shows morphological (**a–d**) and histological (**e–h**) resemblance to both parents. (**a**) Whole specimen (without holdfast). Scale bar: 8 cm. (**b**) First bifurcation of the main stipe. Scale bar: 5 cm. (**c**) Distal stipe showing the frond developmental pattern. Scale bar: 2 cm. (**d**) Sporophyll-like blade with sporangial development (arrow). Scale bar: 3 cm. (**e**) Stipe longitudinal section. Scale bar: 200 µm. (**f**) Cross section of a vegetative frond. Scale bar: 100 µm. (**g**) Reproductive blade cross section. Scale bar: 100 µm. (**h**) Magnified area from (**g**) revealing the sorus area in the sporophyll. Scale bar: 40 µm. Mu: mucilage duct, Me: medulla; Md: Meristoderm, Co: cortex, So: Sorus area, Us: unilocular sporangia, Pa: Paraphysis.

The *Macrocystis*-*Lessonia* hybrid was found on a semi-protected rocky platform, in the contact zone between *M. pyrifera* and *L. spicata* (Fig. 1c). At this point of the year (southern hemisphere summer), *M. pyrifera* was the dominant species at Mar Brava, covering over 70% of the littoral zone. *L. spicata* belts were reduced to few scattered adult individuals, most of them with neither blades nor sorus tissue along the remaining thalli (Fig. 1b). The putative hybrid was approximately 1.2 m high and was attached to the rocky platform by a slightly conic holdfast of 15 cm diameter (Fig. 1c). This holdfast comprised unfused haptera of 1.0–1.5 cm diameter. The main stipe close to the holdfast was dark brown, slightly flattened, 2.5 cm thick, hard, with an irregular surface (Fig. 2a,b). This stipe bifurcated repeatedly and became more flexible, cylindrical and light brown in the upper parts; the stipes were ca. 0.7 cm diameter in the distal part of the thalli (Fig. 2c). Corrugated vegetative blades with serrate margins emerged from them, without pneumatocysts at their basis. Some sporophyll-like fronds were also detected close to the thallus base with darker reproductive zones (Fig. 2d) (See Table 1 for more details).

**Table 1:** Summary of external and histological features of *Macrocystis pyrifera*, *Lessonia spicata* and the hybrid between them found at Mar Brava, Chiloe. SD = Standard deviation.

Holdfast	M. pyrifera^*^	L. spicata^*^	Hybrid^*^
Hapteria organization	Branched - unfused	Fused	Branched - unfused
Hapteron diameter (cm)	0.34 (0.07)	—	1.13 (0.21)
Holdfast diameter (cm)	12.75 (2.92)	13.98 (2.70)	16.37
**Stipe**			
Main stipe sturdiness	Flexible	Semi-rigid	Semi-rigid
Stipe shape	Cylindrical	Cylindrical/flat	Cylindrical/flat
Main stipe diameter (cm)	0.97 (0.23)	1.76 (0.36)	2.08
**Fronds**			
Frond shape	Linear to lanceolate	Linear to lanceolate	Linear to lanceolate
Frond margins	Serrate	Entire to slightly denticulate	Entire/Serrate
Frond surface	Corrugated	Smooth	Smooth/Corrugated
Pneumatocysts	Present	Absent	Absent
Sporophylls	Present	Absent	Present
**Internal structure**			
Meristoderm thickness (cell layers)	2–3**	2–3**	2–3
Cortex thickness (cell layers)	10–15**	10–15**	8–10
Medulla thickness (µm)	100**	100**	50–70
Sieve tubes	Present	Absent	Absent
Mucilage ducts	Present	Absent	Present

^*^Based on measurements/observations of 1.2–1.5 m individuals from Mar Brava, Chiloe Island.

**Based on adults (Extracted from *L. nigrescens* Hoffmann & Santelices 1997).

Cross sections through the hybrid stipes, blades and reproductive fronds revealed the presence of three different tissues (Fig. 2e–h). The meristoderm encompassed 2-3 pigmented cell layers, forming a palisade-like parenchyma (visible in Fig. 2h). Contiguously, a cortex tissue was formed by a thick layer of polyhedral unpigmented cells. At the inner section, a medulla mainly comprised a hyphal network, which was entwisted with a longitudinal orientation (Fig. 2f). No sieve tubes were observed between cortex and medulla, but long mucilaginous ducts were abundant in blades and stipes (Fig. 2e,f). Unilocular sporangia were developed in some sporophyll-like fronds, together with unicellular well-pigmented paraphyses (Fig. 2h).

### DNA barcoding

**Figure 3:**
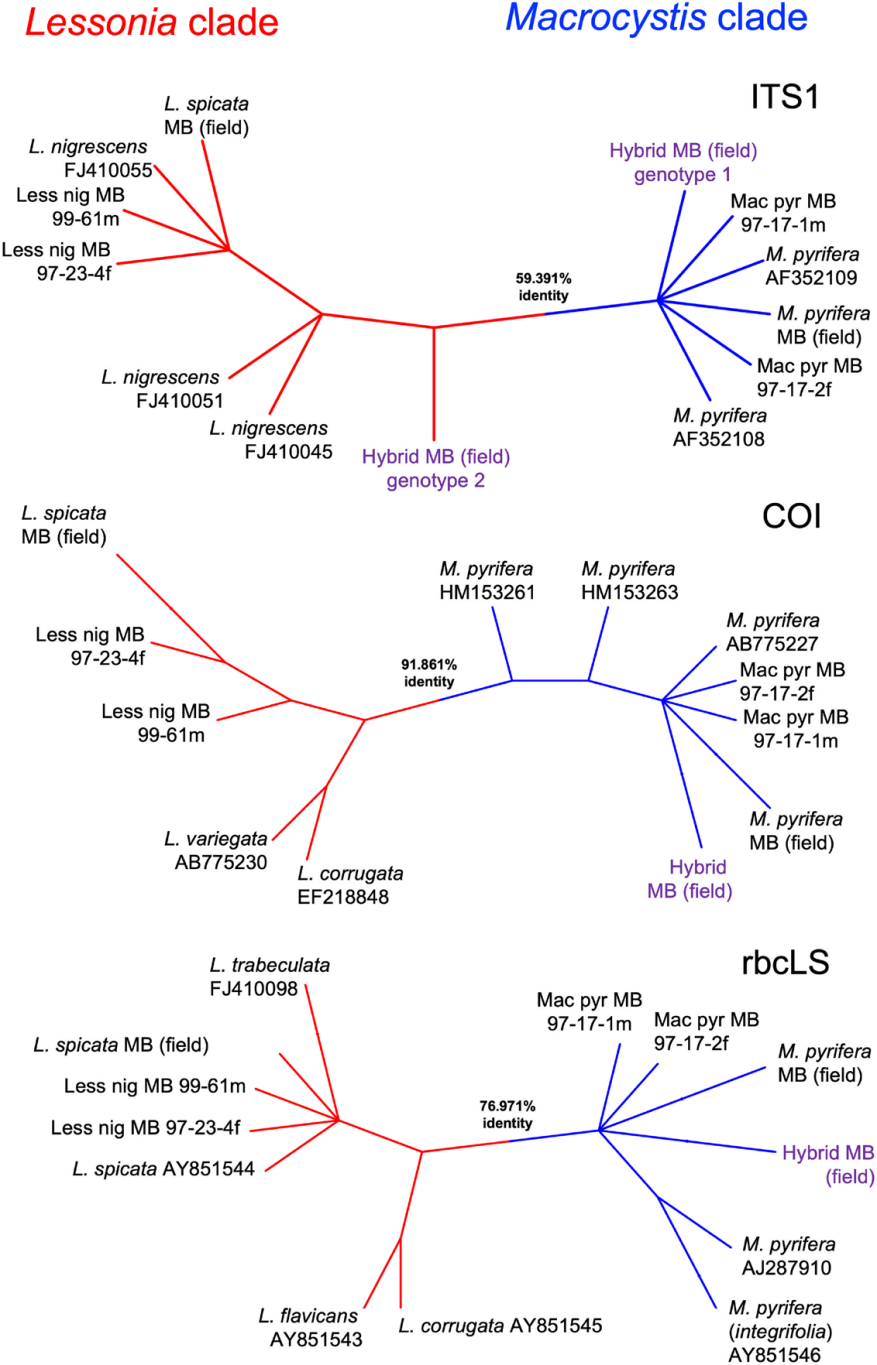
Phylogenetic relationships between *Macrocystis*, *Lessonia* and the wild hybrid. The unrooted trees show the clustering pattern of the field hybrid (purple) with *Lessonia* (red) and *Macrocystis* (blue) lineages using ITS1, COI and *rbc*LS sequences. Average percentage of identity among two clusters are shown in between. For more details on the trees and results from MrBayes and RAxML analyses see Suppl. Figs. 1–3.

Molecular analyses corroborated the presence of two different-sized parental genotypes for ITS1 nrDNA (inset in Fig. 1c). Both agarose gel and sequence results revealed two products of 448 bp and 515 bp allocated in the two bands of the hybrid, similar to putative parental *M. pyrifera* and *L. spicata*, respectively (Fig. 3). The subsequent alignment and phylogenetic reconstruction confirmed that they clustered with the clades of *Macrocystis* and *Lessonia* (Fig. 3; Suppl. Fig. 1). A single band was observed in PCR products of the organellar COI and *rbc*LS spacer markers. The respective sequences clustered only with *Macrocystis* sequences (Fig. 3; Suppl. Figs. 2 and 3).

### Kelp metabolomes

**Figure 4:**
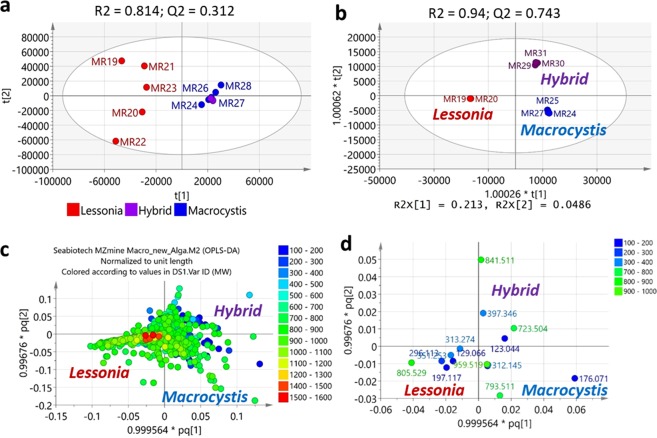
(**a**) Principal Component Analysis (PCA) and (**b**) Orthogonal partial least squares discriminant analysis (OPLS-DA) scores plots of the mass spectral data of collected species of *Lessonia*, *Macrocystis*, and their hybrid. (**c**) OPLS-DA loadings plot indicating the metabolites according to their m/z ion peaks found in the respective species and quadrants designated in b. (**d**) OPLS-DA loadings plot of dereplicated m/z ion peaks listed on Suppl. Table 3.

**Figure 5:**
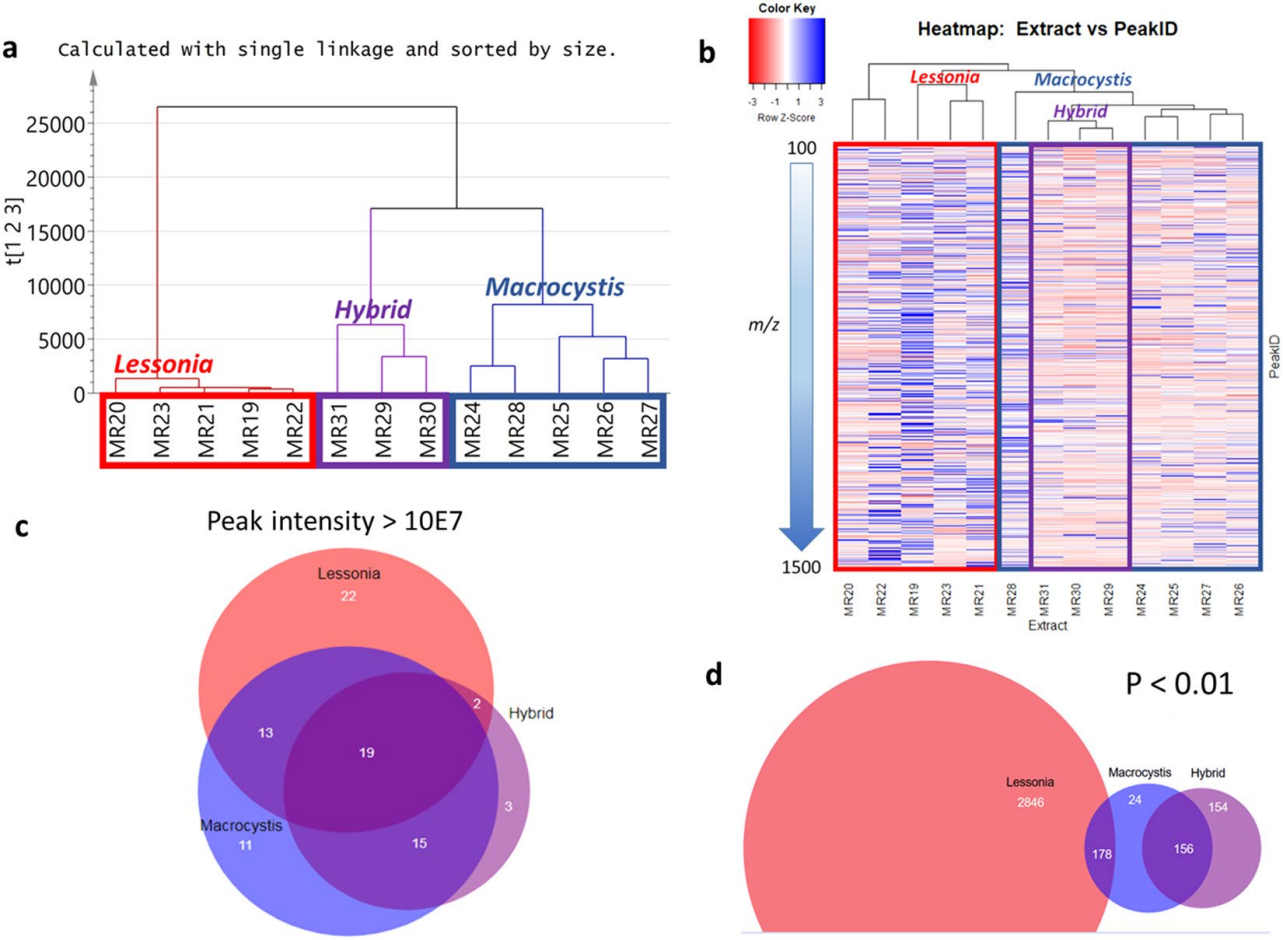
(**a**) Hierarchical Cluster Analysis (HCA) as generated by SIMCA and (**b**) Heatmap dendrogram for the mass spectral data for the collected species of *Lessonia*, *Macrocystis*, and their hybrid. Blue bands indicate increase in relative abundance of metabolites while the decrease in relative abundance of the respective metabolites is designated by pink bands. (**c**) Venn diagram based on average peak intensity >10xE7. (**d**) Venn diagram based on the criteria that the occurrence of the metabolites in the respective regions has a p < 0.01. Dereplication result for common ion peaks in the intersections are presented on Suppl. Table 2 and Suppl. Fig. 5.

Using mass spectrometry, the dataset obtained for the collected species of *Lessonia*, *Macrocystis* and their hybrid was subjected to multivariate analysis (Fig. 4). As shown by Principal Component Analysis (PCA) (Fig. 4a) and Hierarchical Cluster Analysis (HCA) (Fig. 5a) along with a heatmap dendrogram (Fig. 5b), the hybrid was found to be more chemically similar to *Macrocystis* than to *Lessonia*. The predictability score (Q^2^) was quite low at 0.312 due to the observed dispersion of the samples indicating a higher chemical diversity found amongst the *Lessonia* samples in comparison to the clustered samples of *Macrocystis* along with the hybrid. The high chemical diversity of the *Lessonia* samples was also underlined by the higher density of blue bands exhibited by the heatmap shown on Fig. 5b. The Venn diagram based on average peak intensity > 10xE7 (Fig. 5c) from the respective algal strains included 19 mass ion peaks common to *Lessonia*, *Macrocystis* and their hybrid (Suppl. Table 2a and Suppl. Figs. 4 and 5). In parallel, the numbers of overlapping metabolites of the hybrid samples with *Macrocystis* and *Lessonia* strains, which were 15 and 2, respectively, implied that the hybrid had a metabolome more similar to *Macrocystis*. On the other hand, the Venn diagram based on p < 0.01 (Fig. 5d) was more compatible to the dendrograms created from the HCA (Fig. 5a) and the heatmap (Fig. 5b). In such cases there were no overlapping mass ion peaks between *Lessonia* and the hybrid. The intersection between *Lessonia* and *Macrocystis* samples shared 178 mass ion peaks while the hybrid yielded 156 similar ion peaks with the *Macrocystis* samples. Interestingly, 154 peaks were exclusively present in the hybrid (Fig. 5d). Only the top 25 most intense ion peaks have been listed on Suppl. Tables 2b (*Lessonia* x *Macrocystis*) and 2c (*Macrocystis* x hybrid).

An orthogonal partial least squares discriminant analysis (OPLS-DA) was performed to identify the discriminating metabolites that would separate the three sample groups at p < 0.01 (Fig. 4b,c), which were highlighted in grey on Suppl. Table 3. The fitness and predictability scores were at R^2^ = 0.94; Q^2^ = 0.743, respectively, which were typical for metabolomic models using spectral-based datasets^59^. Since the difference between R^2^ and Q^2^ is less than 0.30, the model is confident not to be over fitted. The model is also considered valid from the permutation test resulting to a Q^2^Y intercept <0 at −0.11. The percentage variation between classes was 21.3% while the internodal variation was only 4.91% demonstrating very good similarity and clustering of the samples within their respective classes.

### F2 generation cultivation

**Figure 6:**
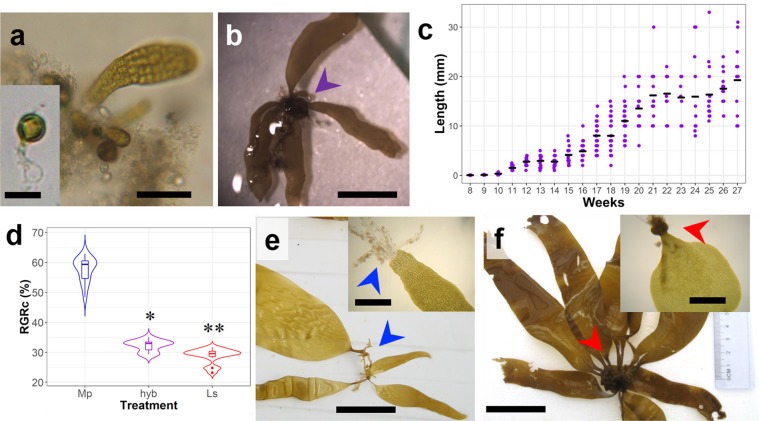
Development of the wild *Macrocystis* x *Lessonia* progeny in culture. (**a**) Emergence of early embryo from unicellular to few-celled female gametophytes. Scale bar: 14 µm. Inset: Germination pattern of the hybrid spores showing an empty embryospore and a primary cell of the gametophyte. Scale bar 8 µm. (**b**) Juvenile individual with fused-haptera holdfast (arrowhead). Scale bar: 1 cm. (**c**) Growth tendency of the hybrid progeny until 27 weeks, time point where they all suddenly died off. Segment = mean. (**d**) Corrected relative growth rate (RGRc) for the progeny from *M. pyrifera* (Mp), *L. spicata* (Ls) and the field hybrid (hyb). Boxes show median (horizontal bold line) ± 1.5 times the interquartile range (whiskers). Dots represent deemed outliers, and asterisks represent statistical groups after a Tukey’s test (p < 0.05). (**e,f**) Morphology of juvenile *M. pyrifera* (**e**) and *L. spicata* (**f**) from Mar Brava after 40 weeks, showing their characteristic holdfast morphology (arrowheads). Scale bars: 2.5 cm. Insets: holdfast morphology of the same species at 15 weeks. Scale bars: 350 µm.

From the hybrid reproductive tissue, a culture was initiated. Settled spores germinated within the first five days (inset on Fig. 6a and entered gametogenesis; female spores formed an oogonium after one to few-cell vegetative development. Early embryos appeared after four weeks (Fig. 6a). Usually, juvenile sporophytes showed a high variability in terms of size and weight. During 27 weeks of culture (Fig. 6b,c), they grew at 30% per week, significantly slower that a culture of *M. pyrifera* from Mar Brava but slightly faster than a *L. spicata* from the same locality (Fig. 6d). They had a typical *Lessonia*-like developmental pattern (Fig. 6b; compare with developmental pattern of *M. pyrifera* and *L. spicata* in Fig. 6e,f), starting to thick the only blade and holdfast from the 15^th^ week onwards. During the 27^th^ week (individuals of 19 mm cm and 31 mg FW; Fig. 6c) sporophytes died off massively, making impossible subsequent analysis.

### *In-vitro* hybridization success

**Figure 7:**
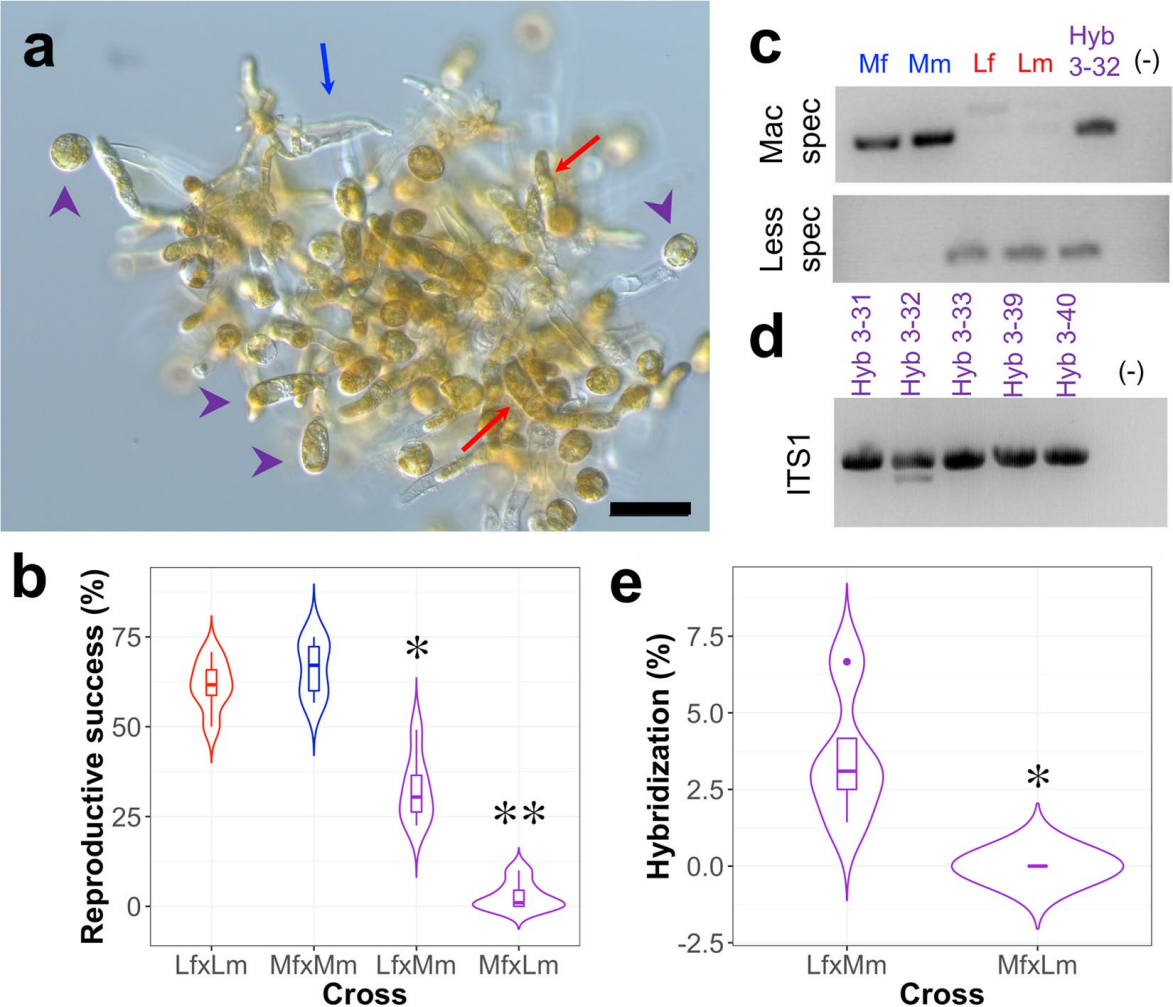
*In vitro* interfamilial hybridization of *Macrocystis pyrifera* and *Lessonia spicata*. (**a**) Crossing outcome of *L. spicata* female gametophytes (Lf, red arrows) and *M. pyrifera* male gametophytes (Mm, blue arrow) highlighting the presence of released eggs cells (putatively their hybrids) (purple). Scale bar: 50 µm. (**b**) Percentage of reproductive success (based on the ratio between released eggs and early embryos per single female gametophyte) of intraspecific and interfamilial crosses between *M. pyrifera* and *L. spicata*. Boxes show median (horizontal bold line) ± 1.5 times the interquartile range (whiskers) and asterisks represent statistical groups after a Tukey’s test (p < 0.05) (to see strain details see Suppl. Table 1). (**c**) Example of amplicon patterns using specific primers for *M. pyrifera* and *L. spicata* in unispecific strains and hybrids from Lf x Mm (Hyb 3-32). (**d**) On the same hybrid, a double band using generic ITS1 markers is also observed. When patterns from (**c,d**) altogether were not observed, such individuals were disregarded as true hybrids and they were assumed as apomictic artefacts. (**e**) Comparison of actual hybridization percentage between reciprocal *L. spicata* x *M. pyrifera* crosses using species-specific and the generic ITS1 markers. Boxes show median (horizontal bold line) ± 1.5 times the interquartile range (whiskers). Dots represent deemed outliers and asterisks represent statistical groups after a Tukey’s test (p < 0.05).

Interfamilial crosses were attempted in order to check the extent of hybridization in laboratory conditions, using clonal *M. pyrifera* and *L. spicata* gametophyte stocks from Mar Brava. The reproductive success (RS) of all combinations was assessed by the quantification of released egg cells per female gametophyte, as exemplified in Fig. 7a. Whereas the RS in intraspecific crosses was above 60%, the interfamilial cross Lf x Mm dropped to ca. 30% and the reciprocal Mf x Lm almost zero (p < 0.05) (Fig. 7b) but cultures were maintained. The sporophytes resulting from released eggs were checked for hybridization using species-specific (Fig. 7c) and generic primers (Fig. 7d), both amplifying the ITS1 region. These three primers were consistent and revealed that most of the resulting sporophytes were apomictic, with only 2.6% true hybrids in the Lf x Mm cross and no hybridization detectable in Mf x Lm (Fig. 7e).

## Discussion

### First report of an interfamilial hybrid in brown algae

Photosynthetic stramenopiles (a.k.a. heterokonts) constitute at least eleven distinct lineages, including some of the most important and abundant algae like the Phaeophycean order Laminariales^60^. Within them, kelps diverged from Ectocarpales and less-related brown algae after the brown algal crown radiation^61^. From studies started by Lane *et al*.^33^ and later confirmed with different phylogenomic approaches, we currently know that *Macrocystis* and *Lessonia* belong to different clades within the Laminariales, distant enough to separate them in the two different families Arthrothamnaceae and Lessoniaceae. The genetic distance between both genera (e.g., ca. 8% for COI) is much larger than we would normally expect between fully compatible interfertile species (i.e. species cut-off in *Macrocystis* is 1.8% in the same gene, Macaya *et al*.^16^). Natural hybrids in the Fucales, for instance *Fucus serratus* x *F. distichus*, show only 1% difference in this gene^26^. Likewise, natural hybrids between *Ectocarpus* species (e.g. *siliculosus* x *crouaniorum*), with a genetic distance of 5% in COI, already show inhibition of meiosis^31^. In Desmarestiales, COI pairwise distance starts at 2.4%^62^. This situation draws questions about the genetic and genomic mechanisms that inhibit interfertility between such different species. Meiosis seems to lead to at least initially viable offspring in our adult *Lessonia-Macrocystis* hybrid, suggesting that maybe the number of chromosomes does not differ between these kelps.

### Wild versus laboratory-induced hybridization in brown algae

Natural hybridization in brown algae is considered atypical in field studies, especially with viable offspring. Coyer *et al*.^63^ published the first case of a fertile kelp hybrid (*Macrocystis*-*Pelagophycus*) in nature. Hybrid gametophytes were diploid, carrying a full set of both parental chromosomes, which may have allowed meiosis and subsequent sexual reproduction^64^. Fertile hybrids from two *Fucus* species (*F. serratus* x *F. distichus*) have also been reported within a secondary contact zones^26^, following an accidental introduction in the Baltic. Most of hybrids reported for Laminariales and Ectocarpales have shown full sterility, likely as product of either abortive sporangia or sterile gametophytes after chromosome mismatches^65–67^. In *Ectocarpus*, Montecinos *et al*.^31^ demonstrated high rates of aneuploidy and expression of rare alleles in diploid sporophytes and absence of haploid recombinant *Ectocarpus* hybrids (*E. siliculosus* x *E. crouaniorum*) in the field, reinforcing the hypothesis that anomalous chromosome segregation during meiosis and subsequent hybridization (of different size genomes) might be a major cause of interspecific incompatibility for these species. The absence of interfamilial hybrids in nature between these species (inc. *Macrocystis* x *Lessonia*) is probably regulated by strong pre- and post-zygotic barriers. As those described in plants, the pre-zygotic barriers may be linked to chromosome translocation differences between the parent species or genetic determinism of sterility (e.g. gene(s) highly expressed majorly in hybrids). This may explain the very low generation of hybrids in sexually compatible interfamilial strains and the low pervasiveness of such hybrids in later stages. In nature, secondary contact zones between both species are rather unusual in the normally *Lessonia*-dominated intertidal^68^, where its coexistence and hybridization may be influenced by environmental (e.g. substrate availability) or anthropogenic (*L. spicata* abundance decrease by overharvesting) interactions. Alternatively, it is unknown whether the hybrid we observed corresponds to an extremely rare event or perhaps a more common phenomenon, although perhaps hidden by one parental phenotype.

In laboratory experiments hybrid offspring has been much easier to obtain^28^, and there are many records for kelps such as *Laminaria*, *Alaria*, *Undaria* and *Macrocystis*^69–73^. Several of these studies, nevertheless, assumed hybridization uniquely by morphological (sporophyte malformations) and developmental changes (egg cell release from the female oogonium) that may lead to mating (e.g. reproductive success) overestimation. Members of the Laminariales (kelps) share several specific reproductive traits: heteromorphic life cycle with alternation between a minute/filamentous –typically haploid- gametophyte and a much larger parenchymatous diploid sporophyte and oogamous sexual reproduction. Sperm release from antheridia and subsequent attraction to released eggs is mediated by the same pheromone, lamoxirene^74,75^. Interspecific crosses within Laminariales seemed biologically possible because of the conservation of their mating systems, same sex hormone and overlapping phenological requirements for gametogenesis^76^. Nevertheless, in our study the success of *in-vitro* hybridization was rather low and masked by apogamy. Hoarau *et al*.^77^ determined that the sexual compatibility between two *Fucus* species is inversely correlated with their sympatry, which may explain the low interfamilial mating success in our laboratory experiments using only Mar Brava reciprocal crosses. We need to expand the number of parents to other more distant localities and test if this response is consistent for *Macrocystis* x *Lessonia* hybridization.

### Metabolomics as a tool to unravel hybridization signatures as well as to indicate the occurrence of putative novel compounds in kelps

A metabolome-wide screening was used to check whether there were compounds shared across our kelps. This study revealed that whilst the wild hybrid is more comparable to *Macrocystis*, a few compounds with peak intensity >10E7 are shared exclusively with *Lessonia*. More interestingly, there are 3 and 54 metabolites potentially exclusively expressed in the hybrid in terms of their peak intensity and significance (P < 0.01), respectively. This includes 4-hydroxybenzaldehyde (compound 20 in Suppl. Fig. 4), earlier described from the chlorophyte green alga *Boodlea composita*^78^ for the ion peak at *m/z* 123.0438 [M + H]. Another ion peak at *m/z* 282.2065 [M + H] was putatively identified either as homoserine lactone (compound 21, Suppl. Fig. 4), previously isolated from a marine-derived *Mesorhizobium* sp. strain R8-Ret-T53-13d^79^ or scalusamide A (compound 22, Suppl. Fig. 4), also an oxopyrrolidine produced by marine-derived *Penicillium citrinum* strain N 055^80^. Other metabolites detected included non-polar sterols (compounds 22 to 25) earlier reported from the green algae *Bryopsis pennata*^81^ and *Prototheca wickerhamii*^82^. Similar to the *Macrocystis* samples, the occurrence of glycosyl glycerides was also observed. The detected glycerides 26 and 28 (Suppl. Fig. 4) were first described from the red alga *Gigartina tenella*^83^ and the green alga *Chlorella vulgaris*^84,85^, respectively. Phaeophytin (27), a chlorophyll breakdown product, was also identified for the ion peak at *m/z* 901.5839 [M + H]^86^. While several of them may be products from the associated microbiome, their exclusivity in the hybrid raise questions about differential microbiome composition in the hybrids in comparison of its parental kelps or alternative metabolic pathways linked to intermediate metabolomes.

Overall, *Lessonia* species were dominated by unknown higher MW compounds at 700-1000 Da with a higher density of metabolites >1000 Da (red dots in Fig. 4c), when compared to *Macrocystis* and the hybrid samples that had lower MW compounds (ca. 100-400 Da). Using the Dictionary of Natural Products (version 2019), a dereplication study accomplished the putative identification of 48 algal and marine-derived natural products, which were found to be featured in the three strains being investigated in this study (Suppl. Tables 2 and 3 and Suppl. Figs. 4 and 5). Amongst the dereplicated metabolites, 18 compounds have been earlier described from algal sources (Fig. 4d and Suppl. Table 2), and three specifically from kelps. The discriminating metabolites identified for the respective strains were mostly from marine-derived microbial sources. Most of the discriminating and common metabolites for the three strains were found to be unsaturated *N*-containing compounds. The conserved metabolites for the three strains (Fig. 5c) include peptides and lipopeptides (Suppl. Table 3a; see compounds 30 to 32 in Suppl. Fig. 5). The occurrence of unidentified peptides was evident by the almost equal ratios of nitrogen to oxygen and double bond equivalence (DBE) while the molecular weight is approximately 100x of the DBE^54^ (Suppl. Table 3). The kelp metabolite loliolide (compound 1 in Suppl. Fig. 4) was also detected amongst the 19 common metabolites along with another low molecular weight compound, cladoacetal A (compound 33 in Suppl. Fig. 5), earlier isolated from the marine-derived fungi *Pestalotiopsis vaccinii* cgmcc3.9199^87^ and *P. heterocornis* XWS03F09^88^. The Venn diagram based on P < 0.01 (Fig. 5d) also indicated the higher occurrence of olefinic and alkyl compounds 34 and 38 to 41 in *Macrocystis* and *Lessonia* samples (Suppl. Table 2b and Suppl. Fig. 5). This could be fatty acids, glycerides, or short chain lipids exhibiting a 2:1 ratio of hydrogen to the carbons and with low DBE.

Amongst the reported sources of the dereplicated hits, we only found hits similar to *Undaria pinnatifida*. Glycosyl glycerides along with polar lipids, and free fatty acids have been commonly described in these kelps^89^. In addition, the occurrence of auxin 1*H*-indole-3-acetic acid (IAA) and its analogues play an important role in kelp’s environmental polarization in response to gravity and light vectors^90,91^. Earlier reported metabolites (compounds 1, 4b, and 19 in Suppl. Fig. from the kelp *U. pinnatifida* were only detected in the *Macrocystis* and *Lessonia* samples but not in the hybrid. From the metabolomics profile data of the dereplicated compounds, the presence of glycosyl glycerides was also common between *Macrocystis* and the hybrid (Suppl. Table 2b,c). The dereplicated hits for mass ion peaks perceived in the Venn diagram’s intersecting region between *Macrocystis* and the hybrid (Suppl. Table 2c and Suppl. Fig. 5) counted in a higher prevalence of alkaloidal compounds 13 to 15, 21 and 22, 42 to 48, which have been previously isolated from marine-derived fungi. Whereas, suggested alkaloid “hits” not reported from marine-derived sources could imply the presence of new natural products that are yet to be studied.

### Considerations of the Macrocystis/Lessonia model for kelp interfamilial hybridization studies

The implications from this study are not only ecological but also potentially practical (e.g. aquaculture). Hybrids from two genetically different kelps often are good candidates to generate cultures with intermediate features^14^, although some negative aspects must be considered such as cross-fertilization with natural stocks^92^ leading to genetic pollution or invasive genotype emergence. Individual genetic heterogeneity is proven to increase fitness in natural populations^43^. Kelp hybridization has been proposed as a strategy to increase commercially relevant traits in optimized maricultures as a result of the heterosis effect^69,93^. It can potentially improve parental attributes, although gene flow/introgression has never been assessed in this context as in red seaweeds^29^, which may constitute a potential weakness. *Lessonia/Macrocystis* interfamilial crosses from the Mar Brava population showed low compatibility in this study, but these results may be expanded as gametophytes with geographically distant origins are used, and productivities or other traits are optimized as it has been demonstrated for brown seaweeds^77^ and plants^94^.

Our results confirmed the interfamilial hybridization between the kelp genera *Macrocystis* and *Lessonia*, both *in situ* and in laboratory conditions. A wild adult hybrid, which morphologically resembled both parents, naturally recruited in a *Lessonia*-dominated habitat. This hybrid was fertile, and its progeny grew until several mm in size before dying. These events suggest no chromosomes mismatches during meiosis and fertilization. With species-specific markers we detected that this hybridization might be reciprocally viable. Nevertheless, since not all gametophyte combinations hybridized in our crosses (asymmetric sexual selection), the studied interfamilial sexual compatibility may be genetically determined. It seems that due to pre- and post-zygotic barriers (e.g. hybrid breakdown) the hybridization success is rather low. Additionally, hybrids seem easily overgrown by pseudogamy-derived parthenosporophytes when gametophytes from both species interact. This would explain why it is an unusual and overlooked event in nature. We provided fundamental evidence that kelp interfamilial hybridization may potentially happen in the field and can be obtained in the laboratory. This information may be relevant to understand the population dynamics of both founding species at community level, but also to understand the latent aquaculture fingerprint of future kelp breeding programs.

## Supplementary information

Supplementary information.
