# Publisher Correction: Morphological, genotypic and metabolomic signatures confirm interfamilial hybridization between the ubiquitous kelps *Macrocystis* (Arthrothamnaceae) and *Lessonia* (Lessoniaceae)

**DOI:** 10.1038/s41598-020-68820-7

**Published:** 2020-07-14

**Authors:** Pedro Murúa, RuAngelie Edrada-Ebel, Liliana Muñoz, Sylvia Soldatou, Nathalie Legrave, Dieter G. Müller, David J. Patiño, Pieter van West, Frithjof C. Küpper, Renato Westermeier, Rainer Ebel, Akira F. Peters

**Affiliations:** 1 Instituto de Acuicultura, Universidad Austral de Chile, Sede Puerto Montt, PO Box 1327, Puerto Montt, Chile; 2 The Scottish Association for Marine Science, Scottish Marine Institute, Culture Collection for Algae and Protozoa, Oban, Argyll PA37 1QA Scotland, UK; 3 Aberdeen Oomycete Group, College of Life Sciences and Medicine, University of Aberdeen, Foresterhill, AB25 2ZD Aberdeen UK; 4 The Natural Products Metabolomics Group, Strathclyde Institute of Pharmacy and Biomedical Sciences, University of Strathclyde, The John Arbuthnott Building, 161 Cathedral Street, Glasgow, G4 0RE UK; 5 Marine Biodiscovery Centre, Department of Chemistry, University of Aberdeen, Meston Building, Meston Walk, Old Aberdeen, AB24 3UE UK; 6 Fachbereich Biologie der Universität Konstanz, D-78457 Konstanz, Germany; 7 School of Biological Sciences, University of Aberdeen, Cruickshank Building, St Machar Drive, Aberdeen, AB24 3UU Scotland, UK; 8 Bezhin Rosko, 40 rue des pêcheurs, 29250 Santec, Brittany France

Correction to: *Scientific Reports* 10.1038/s41598-020-65137-3, published online 19 May 2020

The original version of this Article contained a typographical error in the spelling of the author Pieter van West, which was incorrectly given as Pieter can West. This has now been corrected in the PDF and HTML versions of the Article.

