# Supplementary material for: Morphological, genotypic and metabolomic signatures confirm interfamilial hybridization between the ubiquitous kelps Macrocystis (Arthrothamnaceae) and Lessonia (Lessoniaceae)
Source: Sci Rep. 2020 May 19;10:8279. doi: 10.1038/s41598-020-65137-3 (PMC7237481; doi:10.1038/s41598-020-65137-3)
Supplement: Supplementary file 1 — Supplementary information. [file 41598_2020_65137_MOESM1_ESM.docx]

Supplementary information

**Morphological, genotypic and metabolomic signatures confirm interfamilial hybridization between the ubiquitous kelps *Macrocystis* (Arthrothamnaceae) and *Lessonia* (****Lessoniaceae)**

Pedro Murúa^1, 2, 3^, RuAngelie Edrada-Ebel^4^, Liliana Muñoz^3^, Sylvia Soldatou^5^, Nathalie Legrave^5^, Dieter G. Müller^6^, David J. Patiño^1^, Pieter van West^3^, Frithjof C. Küpper^5, 7^, Renato Westermeier^1^, Rainer Ebel and Akira F. Peters^7, 8^

- Supplementary Figure 1. Bayesian (MrBayes), Maximum likelihood (RaxML and PhyML) and Neighbour-Joining (NJ) cladograms of *Lessonia* and *Macrocystis* lineages and the wild hybrid genotypes from Mar Brava, based on 5’-partial ITS1 rDNA.
- Supplementary Figure 2. Bayesian (MrBayes), Maximum likelihood (RaxML and PhyML) and Neighbour-Joining (NJ) cladograms of *Lessonia* and *Macrocystis* lineages and the wild hybrid genotype from Mar Brava, based on 5’-partial COI mtDNA.
- Supplementary Figure 3. Bayesian (MrBayes), Maximum likelihood (RaxML and PhyML) and Neighbour-Joining (NJ) cladograms of *Lessonia* and *Macrocystis* lineages and the wild hybrid genotype from Mar Brava, based on rbcLS spacer cpDNA.
- Supplementary Figure 4. Structures of putatively dereplicated compounds, specific for *Lessonia, Macrocystis*, and their hybrid
- Supplementary Figure 5. Structures of putatively dereplicated compounds *Lessonia, Macrocystis*, and their hybrid
- Supplementary Figure 6. Unedited and uncropped gel photographs used in this study.
- Supplementary Table 1. List of axenic unisexual strains used in this study.
- Supplementary Table 2. List of dereplicated algal metabolites indicated detected for *Lessonia, Macrocystis*, and their hybrid
- Supplementary Table 3. Intersection mass ion peaks found in the Venn diagrams (for *Lessonia*, *Macrocystis*, and their hybrid


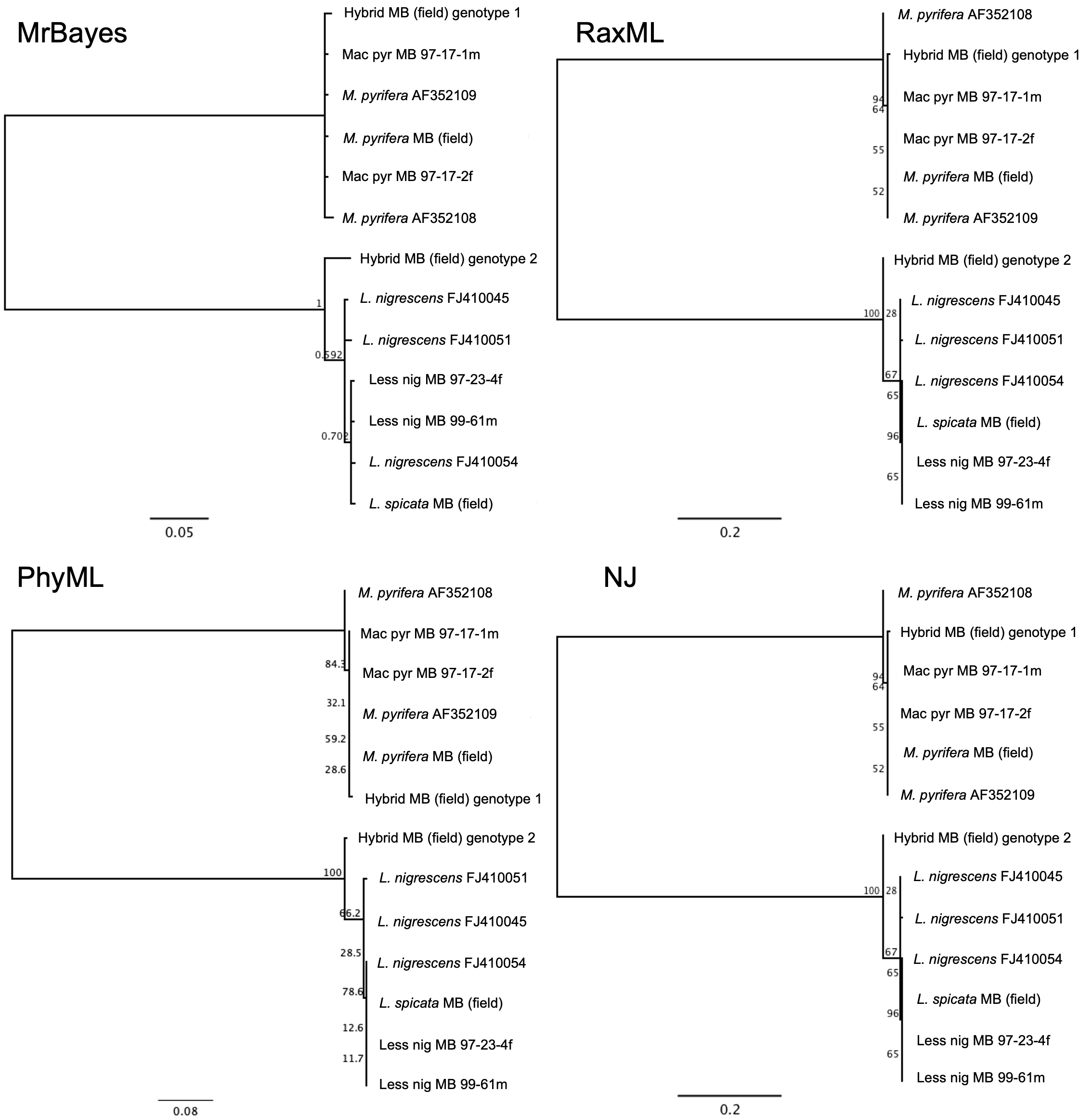


Supplementary Figure 1. Bayesian (MrBayes), Maximum likelihood (RaxML and PhyML) and Neighbour-Joining (NJ) cladograms of *Lessonia* and *Macrocystis* lineages and the wild hybrid genotypes from Mar Brava, based on 5’-partial ITS1 rDNA. These trees contain a total of 13 sequences and 290 positions. Support values correspond to MrBayes posterior probabilities/ML/NJ bootstrap values. The scale bar indicates the number of substitutions per site.


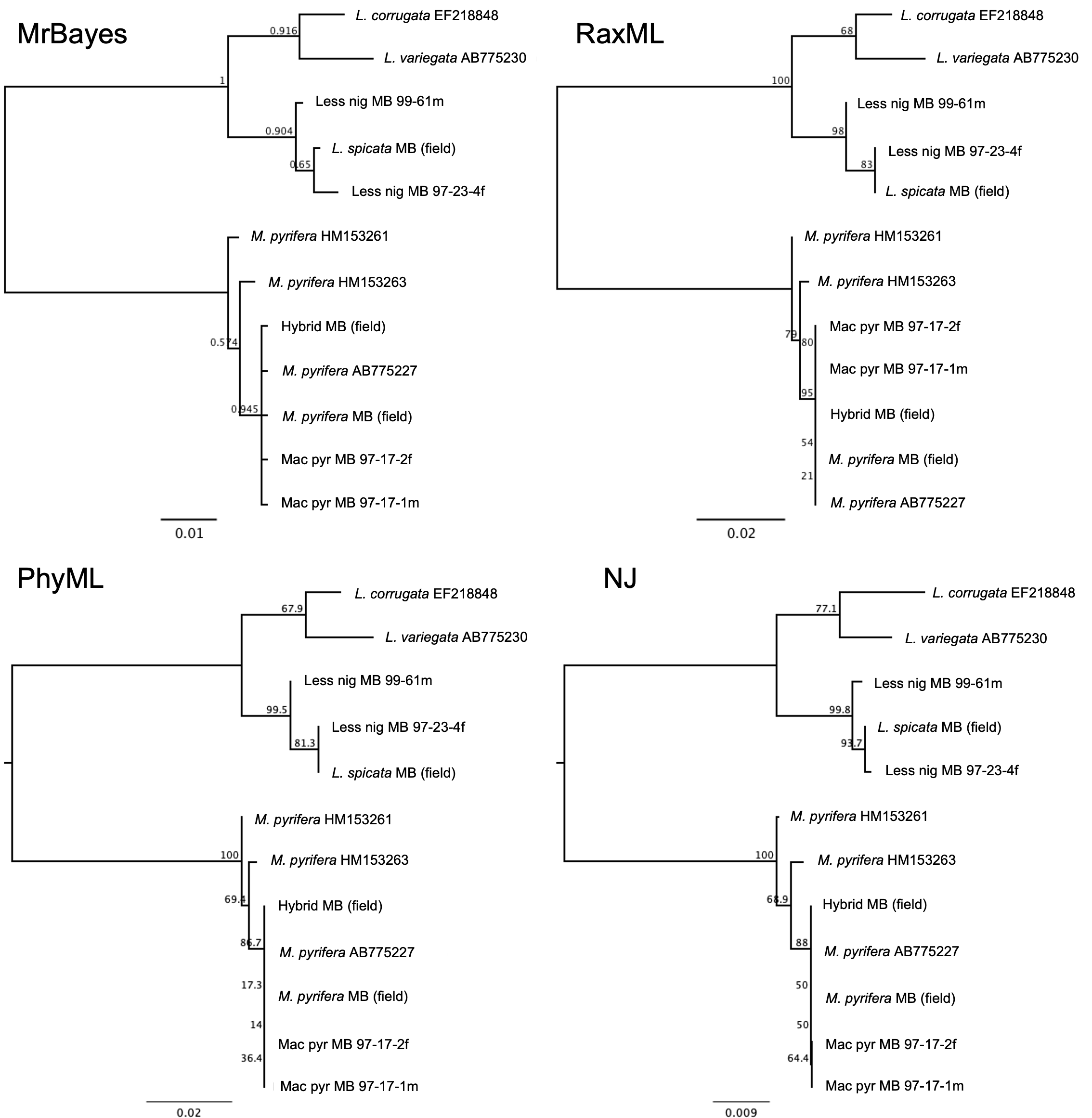


Supplementary Figure 2. Bayesian (MrBayes), Maximum likelihood (RaxML and PhyML) and Neighbour-Joining (NJ) cladograms of *Lessonia* and *Macrocystis* lineages and the wild hybrid genotype from Mar Brava, based on 5’-partial COI mtDNA. These trees contain a total of 12 sequences and 672 positions. Support values correspond to MrBayes posterior probabilities/ML/NJ bootstrap values. The scale bar indicates the number of substitutions per site.


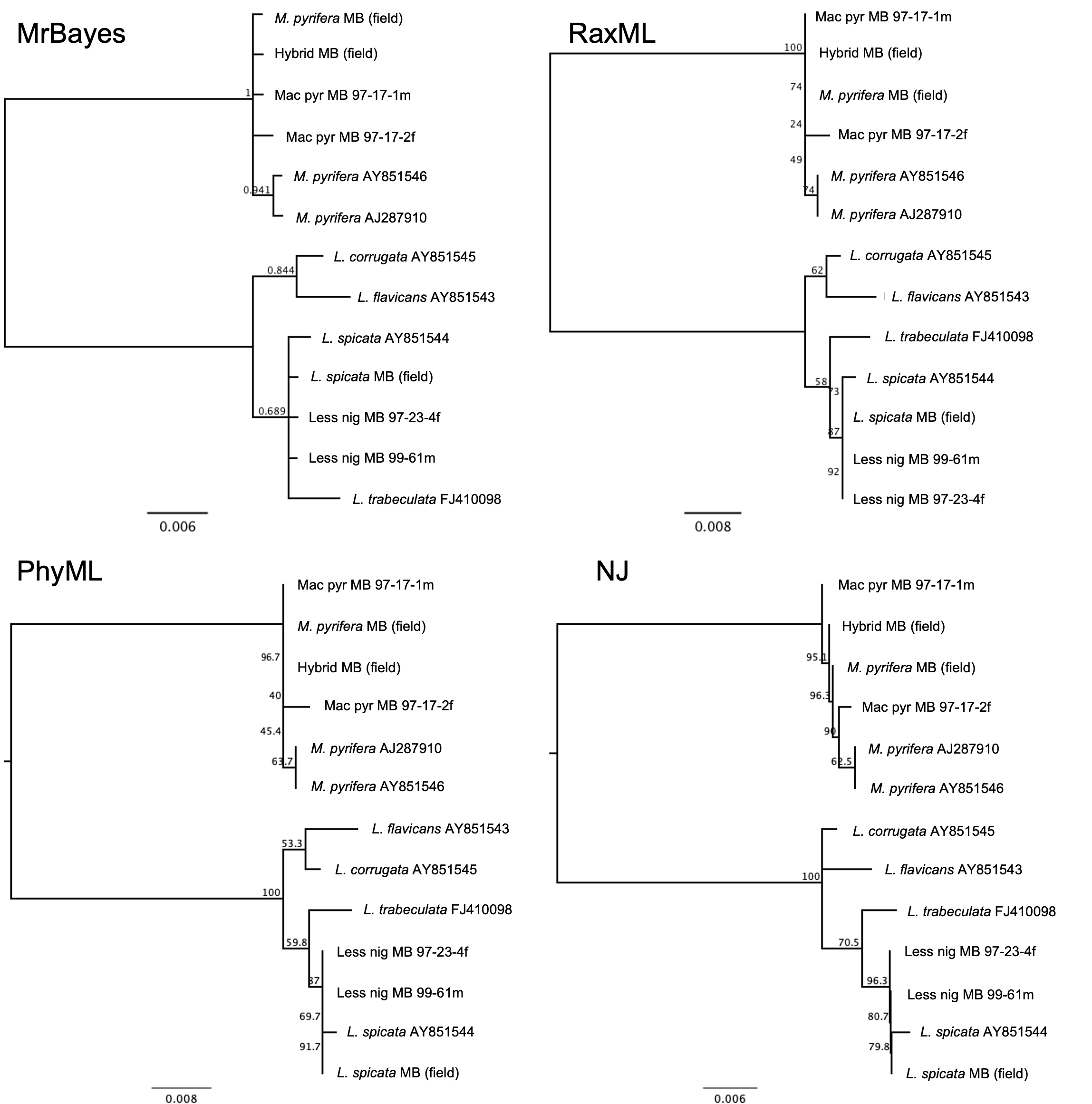


Supplementary Figure 3. Bayesian (MrBayes), Maximum likelihood (RaxML and PhyML) and Neighbour-Joining (NJ) cladograms of *Lessonia* and *Macrocystis* lineages and the wild hybrid genotype from Mar Brava, based on rbcLS spacer cpDNA. These trees contain a total of 13 sequences and 621 positions. Support values correspond to MrBayes posterior probabilities/ML/NJ bootstrap values. The scale bar indicates the number of substitutions per site.

| Supplementary Figure 4. Structures of putatively dereplicated compounds listed on Suppl. Table 2. | | | | | | | | | | | | | | | | | | | |
| --- | --- | --- | --- | --- | --- | --- | --- | --- | --- | --- | --- | --- | --- | --- | --- | --- | --- | --- | --- |
|  |  | | | | |  | | | |  | | | | | |  | | | |
|  |  | | | | |  | | | |  | | | | | |  | | | |
| **1** | **2** | | | | | **3** | | | | **4** | | | | | | **5** | | | |
|  | |  | | | | | | | | | | |  | | | | | | |
| **6** | | **7** | | | | | | | | | | | **8** | | | | | | |
|  | | |  | | | | | | | |  | | | | | |  | | |
| **9** | | | **10** | | | | | | | | **11** | | | | | | **12** | | |
|  | | | |  | | | | | | | |  | | | | | | |  |
| **13** | | | | **14** | | | | | | | | **15** | | | | | | | **16** |
|  | | | | |  | | | | | | | | | | | | | | |
| **17** | | | | | **18** | | | | | | | | | | | | | | |
|  | | | | | | | | | | | | | |  |  | | | | |
| **19** | | | | | | | | | | | | | | **20** | **21** | | | | |
|  | | |  | | | | | |  | | | | | | | | |  | |
| **22** | | | **23** | | | | | | **24** | | | | | | | | | **25** | |
|  | | | | | | |  | | | | | | | | | | | | |
| **26** | | | | | | | **27** | | | | | | | | | | | | |
|  | | | | | | | |  | | | | | | | | | | | |
| **28** | | | | | | | | **29** | | | | | | | | | | | |

| Supplementary Figure 5. Structures of putatively dereplicated compounds listed on Suppl. Table 3. | | | | | | | | | | | | | |
| --- | --- | --- | --- | --- | --- | --- | --- | --- | --- | --- | --- | --- | --- |
|  | | | | | | | |  | | | | | |
| **30** | | | | | | | | **31** | | | | | |
|  | | | | |  | | | |  | | | |  |
| **32** | | | | | **33** | | | | **34** | | | | **35** |
|  | |  | | | | |  | | | | | | |
| **36** | | **37** | | | | | **38** | | | | | | |
|  | | | | | |  | | | | | | | |
| **39** | | | | | | **40** | | | | | | | |
|  | | | |  | | | | | | |  | | |
| **41** | | | | **42** | | | | | | | **43** | | |
|  |  | |  | | | | | | |  | |  | |
| **44** | **45** | | **46** | | | | | | | **47** | | **48** | |


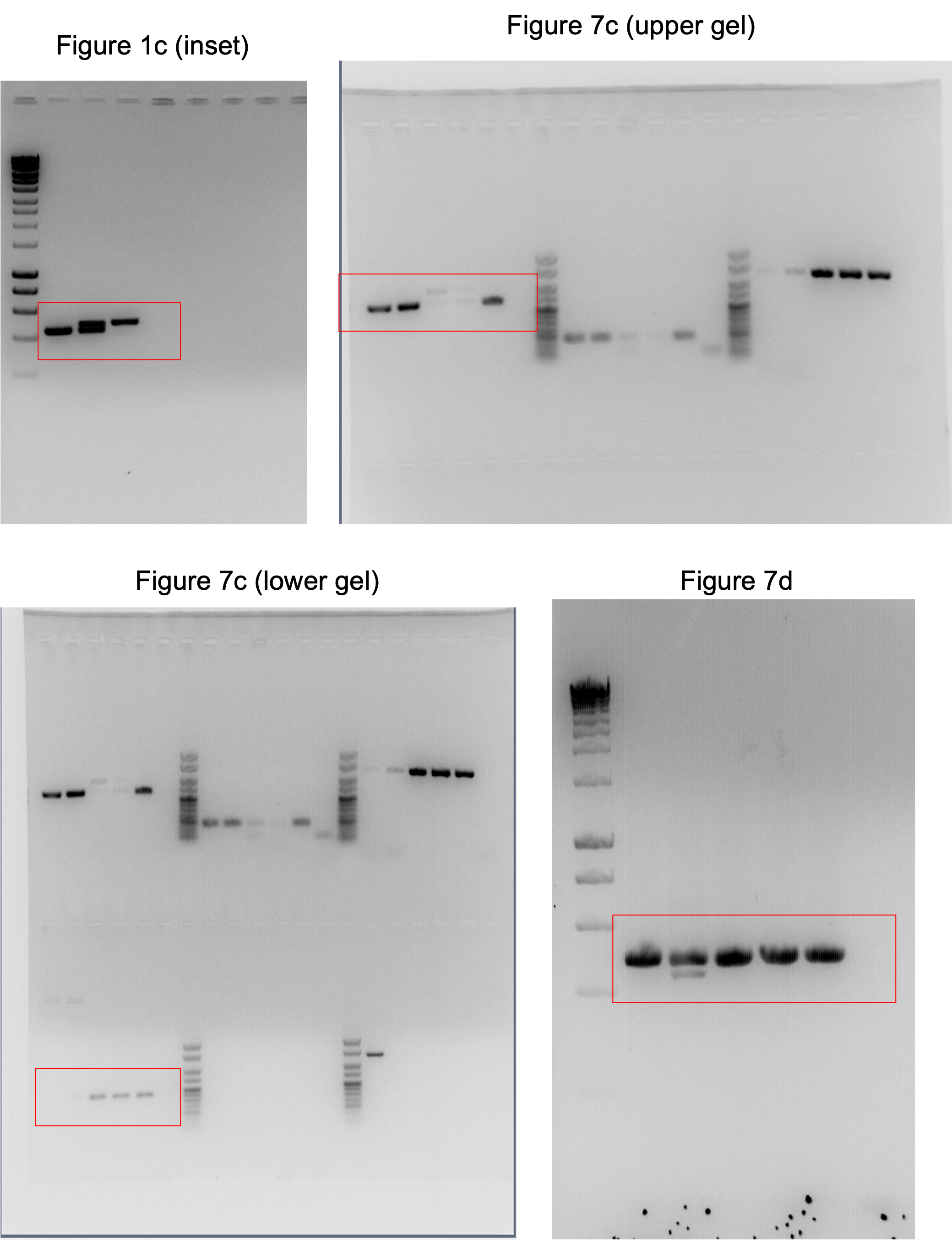


Supplementary Figure 6. Unedited and uncropped gel photographs used in this study. Surrounded in a red frame is the subset used to design the respective plates.

Supplementary Table 1. List of axenic unisexual strains used for DNA barcoding and mating experiments

| Code | Species | Sex | Origin | Sampling Date |
| --- | --- | --- | --- | --- |
| Less nig MB 97-23-4 f (= PM 82) | *L. spicata* | female | Mar Brava, Chiloe | Jan-97 |
| Less nig MB 99-61 m (=PM 84) | *L. spicata* | male | Mar Brava, Chiloe | Sep-99 |
| Mac pyr MB 97-17-2 f (=PM 05) | *M. pyrifera* | female | Mar Brava, Chiloe | Jan-97 |
| Mac pyr MB 97-17-1 m (=PM 06) | *M. pyrifera* | male | Mar Brava, Chiloe | Jan-97 |

Supplementary Table 2. Intersection mass ion peaks found in the Venn diagrams (Figs. 5c and 5d) for *Lessonia*, *Macrocystis*, and their hybrid. *Structures of compound numbered 30 to 45 are shown on Suppl. Fig. 5 while those compounds numbered between 1 and 29 are presented in Suppl. Fig. 4. a) *Lessonia* + Hybrid + *Macrocystis*; b) *Macrocystis* + *Lessonia*; c) Hybrid + *Macrocystis*

|  |  |  | **Average Peak Intensity** | | |  |  |  |
| --- | --- | --- | --- | --- | --- | --- | --- | --- |
| **Var ID** | **m/z**  **[M+H]** | **retention time** | ***Lessonia*** | **hybrid** | ***Macrocystis*** | **Predicted**  **Molecular**  **Formula** | **DBE** | **Dereplicated putative hits***  **(reported source)** |
| 1. ***Lessonia* + Hybrid + *Macrocystis*** (based on average peak intensity 1x10E7, Figure 5c) | | | | | | | | |
| 1 | 468.3080 | 14.17 | 2.56E+07 | 6.43E+07 | 5.18E+07 | C_25_H_37_N_7_O_2_ | 11 | no hits |
| 2 | 141.9580 | 31.40 | 8.51E+07 | 8.10E+07 | 7.77E+07 | no predictions |  |  |
| 4 | 767.5280 | 25.64 | 5.79E+07 | 5.25E+07 | 5.42E+07 | C_39_H_70_N_6_O_9_ | 8 | Me-DL-Chg-DL-Dap-DL-xiThr-Gly-Unk-DL-N(Me)Leu-al (synthesized) |
| 5 | 496.3390 | 16.19 | 6.59E+07 | 4.84E+07 | 3.04E+07 | C_27_H_41_N_7_O_2_ | 11 | alkaloid, no marine-derived hits |
| 7 | 287.8890 | 31.42 | 2.51E+07 | 2.92E+07 | 2.75E+07 | no predictions |  |  |
| 10 | 795.5590 | 27.01 | 6.38E+07 | 2.96E+07 | 2.40E+07 | C_41_H_74_N_6_O_9_ | 8 | no hits (peptide) |
| 12 | 226.9520 | 31.17 | 2.99E+07 | 1.80E+07 | 1.54E+07 | no predictions |  |  |
| 17 | 182.9850 | 31.41 | 2.00E+07 | 2.03E+07 | 1.92E+07 | no predictions |  |  |
| 21 | 385.3170 | 4.40 | 1.79E+07 | 1.07E+07 | 1.25E+07 | C_20_H_40_N_4_O_3_ | 3 | *N*-tetradecanoylarginine (**30**)  environmental DNA expressed in *Escherichia coli* |
| 23 | 197.1170 | 5.99 | 2.29E+07 | 1.79E+07 | 1.32E+07 | C_11_H_16_O_3_ | 4 | loliolide (**1**)  (brown alga *Undaria pinnatifida*) |
| 27 | 751.5330 | 29.75 | 3.64E+07 | 2.04E+07 | 2.35E+07 | C_39_H_70_N_6_O_8_ | 8 | no hits (peptide) |
| 31 | 763.5170 | 30.28 | 1.40E+07 | 1.50E+07 | 1.59E+07 | C_49_H_62_N_8_ | 23 | no hits |
| 34 | 158.9610 | 31.51 | 1.97E+07 | 1.75E+07 | 1.63E+07 | no predictions |  |  |
| 41 | 746.5330 | 23.33 | 1.13E+07 | 2.02E+07 | 2.86E+07 | C_43_H_67_N_7_O_4_ | 14 | no hits |
| 44 | 458.3470 | 13.66 | 1.40E+07 | 1.18E+07 | 1.53E+07 | C_25_H_47_NO_6_ | 3 | 1-acyl-*N*-(2-hydroxydodecanoyl)-2-amino-4-undecene-1,3,6-triol (**31**) |
| 46 | 685.4350 | 30.29 | 1.21E+07 | 1.14E+07 | 1.24E+07 | C_30_H_56_N_10_O_8_ | 8 | no hits (peptide) |
| 86 | 426.3210 | 15.67 | 1.36E+07 | 1.09E+07 | 1.47E+07 | C_24_H_43_NO_5_ | 4 | 2-amino-4-octadecene-1,3-diol derivative (**32**)  (red alga *Amansia glomerata*) |
| 92 | 770.5330 | 23.27 | 1.46E+07 | 1.45E+07 | 1.78E+07 | C_45_H_67_N_7_O_4_ | 16 | no hits |
| 1868 | 205.0860 | 17.23 | 2.11E+07 | 1.07E+07 | 2.12E+07 | C_12_H_12_O_3_ | 7 | cladoacetal A (**33**)  (marine-derived *Pestalotiopsis vaccinii* cgmcc3.9199 and *P. heterocornis* XWS03F09) |
|  |  |  |  |  |  |  |  |  |
| 1. **Macrocystis + Lessonia** (ONLY the top 25 highest intensity peaks were presented out of the 178 common ion peaks based on P<0.01, Figure 5d) | | | | | | | | |
| 39 | 200.9960 | 31.58 | 2.24E+06 |  | 7.39E+06 | no predictions |  |  |
| 58 | 204.9670 | 31.68 | 2.33E+06 |  | 7.12E+06 | no predictions |  |  |
| 93 | 181.1220 | 5.29 | 2.54E+06 |  | 1.34E+06 | C_11_H_16_O_2_ | 4 | 2,4,7-decatrienoic methylester (**34**)  (green alga *Cladophora columbiana*) |
| 109 | 790.6040 | 27.01 | 7.61E+06 |  | 2.74E+06 | C_41_H_71_N_15_O | 14 | no hits |
| 120 | 139.9880 | 31.24 | 9.53E+05 |  | 1.09E+06 | no predictions |  |  |
| 132 | 242.1750 | 6.01 | 2.43E+06 |  | 1.57E+06 | C_13_H_23_NO_3_ | 3 | no hits |
| 158 | 180.1020 | 3.77 | 3.26E+05 |  | 3.48E+06 | C_10_H_13_NO_2_ | 5 | procuramine (**35**)  (marine-derived *Curvularia* sp. IFB-Z1) |
| 215 | 823.5540 | 26.40 | 5.06E+06 |  | 1.45E+06 | C_40_H_62_N_2_0 | 11 | no hits |
| 220 | 825.5690 | 26.84 | 4.32E+06 |  | 8.76E+05 | C_40_H_64_N_2_0 | 10 | no hits |
| 230 | 195.9940 | 31.33 | 1.91E+06 |  | 2.36E+06 | no predictions |  |  |
| 233 | 755.5660 | 27.02 | 4.07E+06 |  | 1.23E+06 | C_41_H_66_N_14_ | 16 | no hits |
| 235 | 954.5780 | 15.76 | 8.47E+06 |  | 1.64E+07 | C_63_H_75_N_3_O_5_ | 28 | no hits |
| 283 | 230.1390 | 6.74 | 2.11E+06 |  | 7.51E+05 | C_11_H_19_NO_4_ | 3 | *N*-tert-butyloxycarbonylhomoproline (**36**)  (red alga *Amphiroa beauvoisii*)  *N*-methyl-(2*S*,5*S*)-pyrrolidinedietylester (**37**)  (red algae *Schizymenia dubyi*  and/or *Haematocelis rubens*) |
| 292 | 848.5640 | 18.37 | 9.69E+05 |  | 4.64E+06 | C_47_H_73_N_7_O_7_ | 15 | no hits |
| 299 | 313.2740 | 27.03 | 3.63E+06 |  | 1.10E+06 | C_19_H_36_O_3_ | 2 | tetrahydro-2*S*-(1*R*-hydroxy-9-nonenyl)-5*R*-pentyl-3*S*-furanol (**38**) (brown alga *Notheia anomala*) |
| 303 | 855.5800 | 25.52 | 4.48E+06 |  | 1.37E+06 | C_43_H_78_N_6_O_11_ | 8 | ngercheumicin G (**39**) (lipopeptide)  (marine-derived *Photobacterium* sp) |
| 306 | 593.5130 | 27.02 | 3.62E+06 |  | 1.16E+06 | C_37_H_68_O_5_ | 4 | α-linoleo-α'-palmitin glyceride (**40**)  (*Oscillatoria* sp) |
| 320 | 793.5430 | 27.11 | 4.49E+06 |  | 8.28E+05 | C_41_H_72_N_6_O_9_ | 9 | no hits |
| 351 | 971.5770 | 13.33 | 2.79E+06 |  | 8.96E+06 | C_57_H_70_N_12_O_3_ | 29 | no hits |
| 368 | 663.5160 | 28.70 | 9.82E+05 |  | 7.68E+05 | C_17_H_29_NO_4_ | 4 | μ-hydroxy-3-isocyano-3-methyloxiranetridecanoic acid (**41**) (*Micromonospora ssp. echinospora*) |
| 395 | 482.3330 | 15.39 | 2.51E+06 |  | 4.14E+05 | C_24_H_43_N_5_O_5_ | 6 | no hits |
| 416 | 818.5980 | 26.39 | 2.00E+06 |  | 5.99E+05 | C_57_H_75_N_3_O | 22 | no hits |
| 417 | 361.7950 | 31.49 | 6.94E+05 |  | 8.26E+05 | no predictions |  |  |
| 425 | 312.2170 | 9.32 | 6.41E+05 |  | 3.29E+05 | C_17_H_29_NO_4_ | 4 | penicillenol D (**42**)  (marine-derived *Trichoderma citrinoviride*) |
| 428 | 1002.6000 | 12.43 | 2.78E+06 |  | 9.18E+06 | C_49_H_75_N_15_O_8_ | 20 | no hits |
| 429 | 522.3550 | 16.73 | 5.24E+06 |  | 9.11E+05 | C_29_H_43_N_7_O_2_ | 12 | no hits |
|  |  |  |  |  |  |  |  |  |
| 1. **Hybrid + Macrocystis** (ONLY the top 25 highest intensity peaks were presented out of the 156 common ion peaks based on P<0.01, Figure 5d) | | | | | | | | |
| 3 | 254.1750 | 4.36 |  | 3.90E+07 | 5.39E+07 | C_14_H_23_NO_3_ | 4 | crilvastatin (**13**) |
| 6 | 318.1700 | 5.00 |  | 2.11E+07 | 2.29E+07 | C_18_H_23_NO_4_ | 8 | sorbicillamine A (**14**)  (marine-derived *Penicillium* sp. F23-2) |
| 8 | 776.5070 | 21.11 |  | 3.26E+07 | 2.97E+07 | C_28_H_61_N_19_O_7_ | 8 | no hits |
| 9 | 159.9690 | 31.56 |  | 2.94E+07 | 2.39E+07 | no predictions |  |  |
| 11 | 282.2070 | 7.03 |  | 1.46E+07 | 7.83E+06 | C_16_H_27_NO_3_ | 4 | *N*-(5*Z*-dodecenoyl)homoserine lactone (**21**) (marine-derived *Mesorhizobium* sp.)  scalusamide A (**22**)  (marine-derived *Penicillium citrinum*) |
| 13 | 268.1910 | 5.31 |  | 1.58E+07 | 2.30E+07 | C_15_H_25_NO_3_ | 4 | 5-oxo-2-pyrrolidine menthyl ester (**15**) |
| 16 | 240.1600 | 3.76 |  | 1.58E+07 | 1.22E+07 | C_13_H_21_NO_3_ | 4 | alkaloid, no marine-derived hits |
| 18 | 302.1750 | 6.44 |  | 1.14E+07 | 1.25E+07 | C_18_H_23_NO_3_ | 8 | alkaloid, no marine-derived hits |
| 19 | 184.9860 | 31.24 |  | 8.43E+06 | 7.82E+06 | no predictions |  |  |
| 29 | 652.4550 | 17.78 |  | 1.03E+07 | 9.18E+06 | C_36_H_57_N_7_O_4_ | 12 | no hits |
| 38 | 304.8920 | 31.49 |  | 1.12E+07 | 1.03E+07 | no predictions |  |  |
| 43 | 226.1440 | 2.93 |  | 1.42E+07 | 2.68E+07 | C_12_H_19_NO_3_ | 6 | alkaloid, no marine-derived hits |
| 50 | 238.1080 | 5.39 |  | 4.22E+06 | 5.30E+06 | C_12_H_15_NO_4_ | 6 | 4-Hydroxy-6-methyl-3-(4-methyl-1,2-dioxopentyl)-2(1H)-pyridinone (**43**)  (marine-derived *Leptosphaerulina* sp) |
| 51 | 265.1550 | 4.43 |  | 4.10E+06 | 8.64E+06 | C_14_H_20_N_2_O_3_ | 6 | penipacid B (**44**)  (marine-derived *Penicillium paneum* SD-44)  bohemamine C (**45**)  (marine-derived *Streptomyces*) |
| 52 | 323.1760 | 5.61 |  | 3.32E+06 | 4.41E+06 | C_20_H_22_N_2_O_2_ | 11 | alkaloid, no marine-derived hits |
| 61 | 762.4920 | 18.97 |  | 5.64E+06 | 4.99E+06 | C_27_H_59_N_19_O_7_ | 8 | no hits |
| 62 | 176.9720 | 31.57 |  | 7.22E+06 | 6.65E+06 | no predictions |  |  |
| 77 | 696.4810 | 20.53 |  | 5.65E+06 | 4.57E+06 | C_23_H_57_N_19_O_6_ | 5 | no hits |
| 78 | 518.3680 | 23.16 |  | 7.18E+06 | 8.44E+06 | C_27_H_51_NO_8_ | 3 | no hits |
| 81 | 280.1550 | 6.94 |  | 2.86E+06 | 1.67E+06 | C_15_H_21_NO_4_ | 6 | 1,6-didehydro- secocycloheximide A (**46**)  (marine-derived *Streptomyces sp.* h-119) |
| 84 | 426.2610 | 14.07 |  | 2.65E+06 | 2.34E+06 | C_22_H_31_N_7_O_2_ | 11 | no hits |
| 85 | 522.3940 | 12.58 |  | 2.56E+06 | 2.09E+06 | C_34_H_51_NO_3_ | 10 | no hits |
| 98 | 296.1130 | 5.45 |  | 2.11E+06 | 1.48E+06 | C_14_H_17_NO_6_ | 7 | *S*- homoglutamic acid (**47**)  (green alga *Ulva rigida*) |
| 99 | 224.0920 | 5.03 |  | 2.92E+06 | 3.45E+06 | C_11_H_13_NO_4_ | 6 | 4,5,6,7-tetrahydro-4,5-dihydroxy-1*H*-indole-3-5-acetylcarboxaldehyde (**48**)  (Puerto Rican *Lyngbya majuscula*) |

| Supplementary Table 3. List of dereplicated algal metabolites indicated in Fig. 4d detected for *Lessonia, Macrocystis*, and their hybrid. *Compound numbers in parenthesis relate to the structures shown below on Suppl. Fig. 4. DBEs (Double Bond Equivalence) were indicated for the unknowns. **Dereplicated metabolites reported from the kelp family. Discriminating metabolites with P < 0.01 shown in Fig. 4d are highlighted in grey. a) *Lessonia; b) Macrocystis; c)* Hybrid*.* | | | | | | | | |
| --- | --- | --- | --- | --- | --- | --- | --- | --- |
| **MZMine ID** | ***m/z***  **[M+H]** | **Retention time (min)** | **MW** | **ppm** | **Dereplicated putative hits**  **(compound no.)*** | **Predicted molecular formula** | **Reported biological source** | |
| 1. ***Lessonia*** | | | | | | | | |
| 3347 | 197.1172 | 3.27 | 196.1099 | -0.23 | loliolide** (**1**) | C_11_H_16_O_3_ | brown alga  *Sargassum crassifolium*  *Undaria pinnatifida*  and red alga  *Gracilaria lemaneiformis* | |
| 3401 | 129.0655 | 3.36 | 128.0582 | -2.62 | squamolone (**2**) | C_5_H_8_N_2_O_2_ | red alga  *Gracilaria lemaneiformis* | |
| 759 | 246.1702 | 3.95 | 245.1629 |  | no hits (DBE = 1) | C_12_H_23_NO_4_ |  | |
| 3124 | 232.1181 | 4.84 | 231.1108 | 0.72 | 1’-hydroxykainic acid (**3**) | C_10_H_17_NO_5_ | red alga  *Digenea simplex*  *Palmaria palmata* | |
| 757 | 263.1393 | 5.10 | 262.1320 |  | no hits (DBE = 7) | C_14_H_18_N_2_O_3_ |  | |
| 98 | 296.1131 | 5.45 | 295.1058 | 0.84 | 1*H*-indole-3-methyl ester (**4A**) | C_10_H_9_NO_2_ | red alga  *Botryocladia leptopoda* | |
| 123 | 227.0817 | 6.82 | 226.0744 |  | no hits (DBE = 10) | C_13_H_10_N_2_O_2_ |  | |
| 2508 | 351.253 | 14.75 | 350.2457 | 0.09 | 9*S*,15ξ-dihydroxy-5*Z*, 7*E*,11*Z*,13*E*-eicosatetraenoic methylester (**5**) | C_21_H_34_O_4_ | red alga  *Polyneura latissima* | |
|  |  |  |  |  | 8,9-epoxy-7-hydroxy-5*Z*, 11*Z*,14*Z*-eicosatrienoic methyester (**6**) | C_21_H_34_O_4_ | red alga  *Polyneura latissima* | |
| 5 | 496.3393 | 16.19 | 495.3320 |  | no hits (DBE = 11) | C_27_H_41_N_7_O_2_ |  | |
| 3239 | 313.274 | 16.38 | 312.2667 | 0.80 | 6,9-Epoxy-18-nonadecene-7,10-diol (**7**) | C_19_H_36_O_3_ | brown alga  *Notheia anomala* | |
| 2392 | 805.529 | 21.93 | 804.5217 | -2.19 | halymecin A (**8**) | C_42_H_76_O_14_ | *Fusarium* sp. FE-71-1 from the marine alga *Halymenia dilatata* | |
| 177 | 814.5595 | 22.66 | 813.5522 |  | no hits (DBE = 9) | C_32_H_67_N_19_O_6_ |  | |
| 10 | 795.5592 | 27.01 | 794.5519 |  | no hits (DBE = 8) | C_41_H_74_N_6_O_9_ |  | |
| 1. ***Macrocystis*** | | | | | | | | |
| 43 | 226.1439 | 2.93 | 225.1366 | 0.56 | no marine-derived hits | C_12_H_19_NO_3_ | | no reported marine sources |
| 3589 | 312.1447 | 3.93 | 311.1374 | 1.76 | domoic acid (**9**) | C_15_H_21_NO_6_ | | red algae  *Chondria armata* and diatoms  *Alsidium corallinum*  *Nitzschia pungens* |
|  |  |  |  |  | isodomoic acid A (**10**) | C_15_H_21_NO_6_ | | diatom  *Nitzschia navis-varingica* |
|  |  |  |  |  | isodomoic acid A; Δ1',1''-isomer (**11**) | C_15_H_21_NO_6_ | | diatom  *Pseudo-nitzschia australis* |
|  |  |  |  |  | isodomoic acid G (**12**) | C_15_H_21_NO_6_ | | red alga  *Chondria armata* |
| 3 | 254.1752 | 4.36 | 253.1679 | 0.66 | crilvastatin (**13**) | C_14_H_23_NO_3_ | | see discussion section |
| 6 | 318.1702 | 4.99 | 317.1629 | 0.61 | sorbicillamine A (**14**) | C_18_H_23_NO_4_ | | marine-derived *Penicillium* sp. F23-2 |
| 13 | 268.1910 | 5.31 | 267.1837 | 0.94 | 5-oxoproline menthyl ester (**15**) | C_15_H_25_NO_3_ | | see discussion section |
| 116 | 176.0705 | 5.98 | 175.0632 | -0.46 | 1*H*-indole-3-acetic acid** (**4B**) | C_10_H_9_NO_2_ | | brown alga  *Undaria pinnatifida* |
| 18 | 302.1753 | 6.44 | 301.1680 | 0.85 | methyl-8-(3-methoxy-3-methylbutyl)-2-methyl-4-quinolinecarboxylate (**16**) | C_18_H_23_NO_3_ | | *Streptomyces sp. neau* 50 |
| 2955 | 176.0704 | 6.72 | 175.0631 | -1.41 | 5-deoxyhyrtiosin A (**17**) | C_10_H_9_NO_2_ | | red alga  *Prionitis lanceolata* |
| 2771 | 793.5113 | 18.87 | 792.504 | -2.19 | 2-O-hexadecanoyl-1-O-(9-hexadecenoyl)glycerol-3-(6-deoxy-6-sulfo-α-D-glucopyranoside) (**18**) | C_41_H_76_O_12_S | | blue-green alga *Phormidium tenue* |
| 128 | 784.5112 | 19.25 | 783.5039 |  | no hits (DBE = 5 ) | C_29_H_65_N_15_O_10_ | |  |
| 2950 | 959.5186 | 22.76 | 958.5114 | -1.46 | 2',3'-dihexadecanoyl- 3-[[(2,3-dihydroxypropoxy)hydroxyphosphinyl]oxy]-2-hydroxypropyl 5-deoxy-5-(dimethylarsinoyl)-β-D-ribofuranoside** (**19**) | C_45_H_88_AsO_14_P | | brown alga  *Undaria pinnatifida* |
| 149 | 768.5161 | 23.05 | 767.5088 |  | no hits (DBE = 5) | C_29_H_65_N_15_O_9_ | |  |
| 41 | 746.5329 | 23.33 | 745.5256 |  | no hits (DBE = 14) | C_43_H_67_N_7_O_4_ | |  |
| 1. **Hybrid** | | | | | | | | |
| 16 | 240.1596 | 3.76 | 239.1523 | 0.59 | no marine-derived hits | C_13_H_21_NO_3_ | | no reported marine sources |
| 3540 | 123.0438 | 4.15 | 122.0365 | -2.33 | 4-hydroxybenzaldehyde (**20**) | C_7_H_6_O_2_ | | chlorophyte alga *Boodlea composita* |
| 11 | 282.2065 | 7.03 | 281.20 | 0.62 | *N*-(5*Z*-dodecenoyl)homoserine lactone (**21**) | C_16_H_27_NO_3_ | | marine-derived *Mesorhizobium* sp. strain R8-Ret-T53-13d |
|  |  |  |  |  | scalusamide A (**22**) | C_16_H_27_NO_3_ | | marine-derived *Penicillium citrinum* strain N 055 |
| 1 | 468.3081 | 14.17 | 467.3008 |  | no hits (DBE = 11) | C_25_H_37_N_7_O_2_ | |  |
| 14 | 326.3783 | 17.87 | 325.3710 |  | no hits (DBE = 0) | C_22_H_47_N | |  |
| 3326 | 397.3464 | 19.79 | 396.3391 | -0.22 | ergosterin (**23**) | C_28_H_44_O | | green alga  *Bryopsis pennata* |
|  |  |  |  |  | 24-methylcholesta-5,7,25-trien-3-ol (**24**) | C_28_H_44_O | | green alga  *Prototheca wickerhamii* |
|  |  |  |  |  | lichesterol (**25**) | C_28_H_44_O | | green alga  *Bryopsis pennata* |
|  |  |  |  |  | ergosterol B1 (**26**) | C_28_H_44_O | | green alga  *Bryopsis pennata* |
| 8 | 776.5073 | 21.10 | 775.5001 |  | no hits (DBE = 8) | C_28_H_61_N_19_O_7_ | |  |
| 2705 | 723.5036 | 23.71 | 722.4963 | -0.76 | glycerol 1,2-di(7*Z*,10*Z*-hexadecadienoate), 3-O-β-D-galactopyranoside (**26**) | C_41_H_70_O_10_ | | green alga  *Chlorella vulgaris* |
| 152 | 901.5839 | 26.49 | 900.5766 |  | phaeophytin (**28**) | C_56_H_76_N_4_O_6_ | | various sources |
| 167 | 923.5655 | 26.74 | 922.5582 |  | no hits (DBE = 25) | C_54_H_70_N_10_O_4_ | |  |
| 1870 | 841.5106 | 31.17 | 840.5033 | -2.87 | (6-sulfoquinovopyranosyl)-(1→3')-1'-(5,8,11,14,17-eicosapentaenoyl)-2'-hexadecanoylglycerol (**29**) | C_45_H_76_O_12_S | | red alga  *Gigartina tenella* |
